# Complexity of the simplest species tree problem

**DOI:** 10.1093/molbev/msab009

**Published:** 2021-01-25

**Authors:** Tianqi Zhu, Ziheng Yang

**Affiliations:** 1 National Center for Mathematics and Interdisciplinary Sciences, Institute of Applied Mathematics, Academy of Mathematics and Systems Science, Chinese Academy of Sciences, Beijing 100190, China; 2 Key Laboratory of Random Complex Structures and Data Science, Academy of Mathematics and Systems Science, Chinese Academy of Sciences, Beijing 100190, China; 3 Department of Genetics, University College London, Gower Street London WC1E 6BT, UK

**Keywords:** concatenation, efficiency, molecular clock, MSC, multispecies coalescent, species tree

## Abstract

The multispecies coalescent model provides a natural framework for species tree estimation accounting for gene-tree conflicts. Although a number of species tree methods under the multispecies coalescent have been suggested and evaluated using simulation, their statistical properties remain poorly understood. Here, we use mathematical analysis aided by computer simulation to examine the identifiability, consistency, and efficiency of different species tree methods in the case of three species and three sequences under the molecular clock. We consider four major species-tree methods including concatenation, two-step, independent-sites maximum likelihood, and maximum likelihood. We develop approximations that predict that the probit transform of the species tree estimation error decreases linearly with the square root of the number of loci. Even in this simplest case, major differences exist among the methods. Full-likelihood methods are considerably more efficient than summary methods such as concatenation and two-step. They also provide estimates of important parameters such as species divergence times and ancestral population sizes,whereas these parameters are not identifiable by summary methods. Our results highlight the need to improve the statistical efficiency of summary methods and the computational efficiency of full likelihood methods of species tree estimation.

## Introduction

The multispecies coalescent (MSC) model (Rannala and Yang 2003) combines the phylogenetic process of species divergences with the population genetic process of coalescent and naturally accommodates “delayed coalescence” (also known as “incomplete lineage sorting,” Maddison 1997), the phenomenon in which gene sequences fail to coalesce in their most recent common ancestor but do so only in more ancient ancestors. Delayed coalescence causes the gene tree for a gene or genomic region to differ from the species tree and is the most important factor for gene-tree–species-tree discordance (Maddison 1997; Nichols 2001; Szöllősi et al. 2015). The MSC provides a natural framework for estimating species trees accounting for genealogical heterogeneity among genes or across the genome (Edwards 2009; Xu and Yang 2016; Kubatko 2019; Rannala et al. 2020).

Two lines of research into the MSC have provided the foundation for species tree methods. The first concerns the probabilities of different gene tree topologies (Hudson 1983; Pamilo and Nei 1988) and algorithms for their efficient calculation given the species tree (Degnan and Salter 2005; Degnan and Rosenberg 2006). The gene tree distribution can be used in the two-step method of species tree estimation, by inferring gene trees for the individual loci and then applying maximum likelihood (ML) to counts of gene tree topologies (as in stells,Wu 2012). Nevertheless, widely used two-step methods, including astral (Mirarab et al. 2014) and mp-est (Liu et al. 2010), are simpler, and estimate species trees for species triplets (assuming the molecular clock) or quartets (without the clock) and then assemble the subtrees to produce a species-tree estimate for all species. Studies of gene-tree probabilities led to the discovery of the “anomaly zone,” the region of the parameter space in which the most probable gene tree has a different topology from the species tree (Degnan and Salter 2005; Degnan and Rosenberg 2006). In the anomaly zone, the two-step method, which uses the most common gene tree as the species tree estimate, will be inconsistent.

The second line of research into MSC is the development of the joint probability distribution of the gene tree and coalescent times (Rannala and Yang 2003). This forms the basis for exact methods of inference, including ML (Yang 2002; Dalquen et al. 2017) and Bayesian methods (Liu and Pearl 2007; Heled and Drummond 2010; Yang and Rannala 2014; Ogilvie et al. 2017; Rannala and Yang 2017). Although heuristic methods use summaries of the data, exact methods use the multilocus sequence alignments directly and naturally accommodate phylogenetic reconstruction errors and uncertainties (Xu and Yang 2016; Kubatko 2019; Rannala et al. 2020).

Simulation has been used to examine the performance of different species-tree methods (e.g., Leaché and Rannala 2011; Mirarab et al. 2014; Chou et al. 2015; Xu and Yang 2016). A limitation of simulation is that it can examine only a small portion of the parameter space and the results often have limited applicability. Analytical results on the efficiency of different methods have been lacking. Here, we analyze species tree estimation under the MSC in the case of three species, with one sequence from each species per locus. We focus on closely related species and assume the JC mutation model (Jukes and Cantor 1969) and the molecular clock. We are in particular interested in the efficiency of the various methods, measured by the probability of recovering the correct species tree.

We consider four inference methods: 1) ML (a full likelihood method under the MSC applied to the multilocus sequence alignments), 2) 2-step (or majority-vote), 3) concatenation (concat), and 4) independent-sites ML (isml, also known as coalescent-aware concatenation or concat) (Xu and Yang 2016). ML is the full-likelihood method and calculates the likelihood function using the multilocus sequence alignments or a sufficient summary. The 2-step method estimates the gene tree at each locus and then uses the most common gene tree as the species tree estimate. It does not account for the uncertainties in the estimated gene trees. For the case of three species considered here, 2-step is equivalent to the maximum pseudolikelihood method (mp-est) (Liu et al. 2010). Concatenation applies ML to the concatenated sequences, assuming that the same tree underlies all sites in the super alignment. In the case considered here, concatenation is equivalent to steac (Liu et al. 2009), which uses average coalescent times over loci as data to infer a gene tree, which is the species tree estimate. Isml (or concat) estimates the species tree by ML under the assumption that all sites, both from the same locus and from different loci, have independent gene trees (Xu and Yang 2016). This was suggested as an improvement to SVDQuartets of Chifman and Kubatko (2014). All four methods considered here use ML, but the likelihood function is applied to different summaries of the same data. Here, we refer to the full-likelihood or full-data method as the ML method, whereas all other methods (2-step, concatenation, and isml) are considered heuristic summary methods: 2-step uses the (estimated) gene tree topologies, whereas concatenation and isml use the site-pattern counts pooled across loci. We derive approximations to the error rate of species tree estimation by the different methods and assess their accuracy. We use the theory to characterize the differences in the use of information in the data by different methods.

## Results

### Multispecies Coalescent in the Case of Three Species

**Fig. 1.**
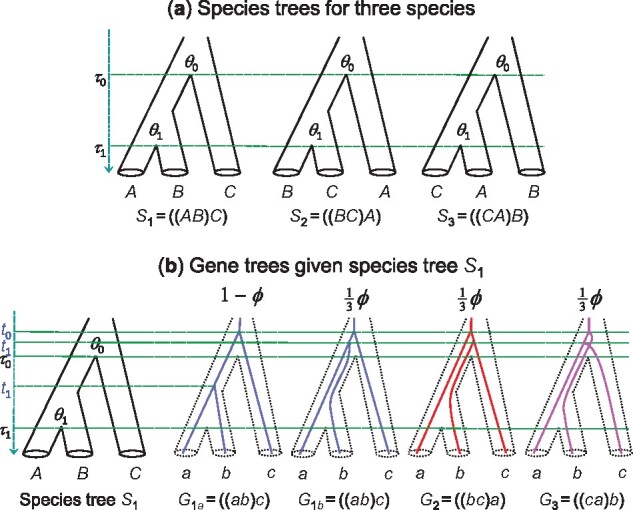
(*a*) The three species trees ($S_{1},S_{2},S_{3}$) for three species (A,B,C) and the parameters in each MSC model. (*b*) The possible gene trees with coalescent times (*t*_0_, *t*_1_) for a locus with three sequences (*a*, *b*, *c*) given the species tree *S*_1_. The probabilities for the gene trees are shown above them, where ϕ = $e^{-\frac{2}{\theta_{1}}(\tau_{0}-\tau_{1})}$ is the probability that *a* and*b* do not coalesce in population *AB* or over the time interval (*τ*_1_, *τ*_0_). Note that if the species tree is *S*_2_ (or *S*_3_), it will be possible for sequences *b* and *c* (or *c* and *a*) to coalesce in the time interval (*τ*_1_, *τ*_0_).

For three species *A*, *B*, and *C*, there are three possible species trees: $S_{1}=((AB)C)$, $S_{2}=((BC)A)$, and $S_{3}=((CA)B)$, each with two divergence times (*τ*_0_ and *τ*_1_) and two population sizes (*θ*_0_ and *θ*_1_) (fig. 1*a*). Both *τ*s and *θ*s are measured by the expected number of mutations per site. For each species, the population size parameter is θ=4Nμ, where *N* is the (effective) population size and *μ* is the mutation rate per site per generation. We consider only one sequence from each species, so that *θ*s for the modern species are not considered. The parameters have different interpretations in different species trees: in *S*_1_, the two ancestral species are *AB* and *ABC* so the parameters are $\theta_{1}$ =  $\left\{\tau_{0},\tau_{1},\theta_{0},\theta_{1}\right\}$  =  $\left\{\tau_{\mathrm{ABC}},\tau_{AB},\theta_{\mathrm{ABC}},\theta_{AB}\right\}$.

At each locus, three sequences (*a*, *b*, and *c*) are sampled, one from each species. They are related through a gene tree. The three possible gene trees are $G_{1}=((ab)c),\;G_{2}=((bc)a)$, and $G_{3}=((ca)b)$, with probabilities:

(1) $$\begin{array}{cc} P(G_{1}|S_{1},\theta_{1}) & =1-\frac{2}{3}\phi ,\\ P(G_{2}|S_{1},\theta_{1}) & =P(G_{3}|S_{1},\theta_{1})=\frac{1}{3}\phi , \end{array}$$

where ϕ =  $e^{-2(\tau_{\mathrm{ABC}}-\tau_{AB})/\theta_{AB}}$ is the probability that sequences *a* and *b* do not coalesce in population *AB* so that all three sequences enter the ancestor *ABC* and the three gene trees occur with equal probability (fig. 1*b*) (Hudson 1983). Here, $2(\tau_{\mathrm{ABC}}-\tau_{AB})/\theta_{AB}$ is known as the internal branch length in coalescent units, as the average coalescent time in population *AB* is $2N_{AB}$ generations or $\theta_{AB}/2$ mutations per site.

For locus *i*, let $t_{i}=\{t_{i0},t_{i1}\}$ be the coalescent times (node ages) on the gene tree (fig. 1*b*). The joint MSC density for the gene tree and coalescent times given species tree *S*_1_ and parameters $\theta_{1}$ is then:

(2) $$\begin{array}{cc} f(G_{1a},t_{i}|S_{1},\theta_{1}) & =\frac{2}{\theta_{1}}e^{-\frac{2}{\theta_{1}}(t_{i1}-\tau_{1})}\cdot \frac{2}{\theta_{0}}e^{-\frac{2}{\theta_{0}}(t_{i0}-\tau_{0})},\\ & \;\;\tau_{1}<t_{i1}<\tau_{0},t_{i0}>\tau_{0},\\ f(G_{k},t_{i}|S_{1},\theta_{1}) & =e^{-\frac{2}{\theta_{1}}(\tau_{0}-\tau_{1})}\\ & \times \frac{2}{\theta_{0}}\frac{2}{\theta_{0}}e^{-\frac{6}{\theta_{0}}(t_{i1}-\tau_{0})-\frac{2}{\theta_{0}}(t_{i0}-t_{i1})},\\ & \;\;t_{i1}>\tau_{0},t_{i0}>t_{i1}, \end{array}$$

for k=1b,2,3 (Takahata et al. 1995; Yang 2002). The probability densities for *S*_2_ and *S*_3_ are given similarly.

The data consist of sequence alignments at *m* loci. Under the JC mutation model, the data at locus *i* can be summarized as counts of five site patterns: *xxx*, *xxy*, *yxx*, *xyx*, and *xyz*, where *x*, *y*, *z* are any three distinct nucleotides. Let those counts be $x_{i}=\{x_{i0},x_{i1},x_{i2},x_{i3},x_{i4}\}$, with $\sum_{j=0}^{4}x_{ij}=n$ to be the number of sites (sequence length) at each locus. Let $f_{ij}=x_{ij}/n$ be the frequencies. Let data at all *m* loci be $x=\{x_{i}\}$.

Given the gene tree and coalescent times at locus *i*, the probability of the sequence data, $f(x_{i}|G_{i},t_{i})$, is given by the multinomial distribution for the five site patterns. For example, given gene tree *G*_1_ with node ages $t_{i0}$ and $t_{i1}$ (fig. 1*b*), the site-pattern probabilities, $p_{i}=\{p_{i0},p_{i1},p_{i2},p_{i3},p_{i4}\}$, are as follows:

(3) $$\begin{array}{c} p_{i0}=P(\mathrm{xxx}|G_{1},t_{i})=\frac{1}{16}(1+3v^{2}+6u+6uv),\\ p_{i1}=P(\mathrm{xxy}|G_{1},t_{i})=\frac{1}{16}(3+9v^{2}-6u-6uv),\\ p_{i2}=P(\mathrm{yxx}|G_{1},t_{i})=\frac{1}{16}(3-3v^{2}+6u-6uv),\\ p_{i3}=P(\mathrm{xyx}|G_{1},t_{i})=p_{2},\\ p_{i4}=P(\mathrm{xyz}|G_{1},t_{i})=\frac{1}{16}(6-6v^{2}-12u+12uv), \end{array}$$

where $u=e^{-8t_{i0}/3}$ and $v=e^{-4t_{i1}/3}$ (Yang 1994b). Note that $p_{i1}>p_{i2}=p_{i3}$ as $t_{i0}>t_{i1}$. The probabilities for gene trees *G*_2_ or *G*_3_ are given by symmetry. Then the sequence data or the five site-pattern counts at the locus have the multinomial probabilities:

(4) $$\begin{array}{c} f(x_{i}|G_{1},t_{i})=p_{i0}^{x_{i0}}p_{i1}^{x_{i1}}p_{i2}^{x_{i2}+x_{i3}}p_{i4}^{x_{i4}},\\ f(x_{i}|G_{2},t_{i})=p_{i0}^{x_{i0}}p_{i1}^{x_{i2}}p_{i2}^{x_{i3}+x_{i1}}p_{i4}^{x_{i4}},\\ f(x_{i}|G_{3},t_{i})=p_{i0}^{x_{i0}}p_{i1}^{x_{i3}}p_{i2}^{x_{i1}+x_{i2}}p_{i4}^{x_{i4}}. \end{array}$$

### The ML Method of Species Tree Estimation

The log-likelihood function for species tree *S*_1_ with parameters $\theta_{1}$ is given by summing over the gene trees and integrating over the coalescent times.

(5) $$\ell_{1}(\theta_{1})=\sum_{i=1}^{m}\;\text{log}\;f(x_{i}|S_{1},\theta_{1})=\sum_{i=1}^{m}\;\text{log}\;\left\{\sum_{G_{i}}\int f(G_{i},t_{i}|S_{1},\theta_{1})f(x_{i}|G_{i},t_{i})dt_{i}\right\},$$

where $f(G_{i},t_{i}|S_{1},\theta_{1})$ is the MSC density for the gene tree and coalescent times at locus *i* (eq. 2), and $f(x_{i}|G_{i},t_{i})$ is the probability of the sequence data at locus *i* given the gene tree (eq. 4). The log likelihood functions, $\ell_{2}(\theta_{2})$and $\ell_{3}(\theta_{3})$, for *S*_2_ (with parameters $\theta_{2}$) and *S*_3_ (with $\theta_{3}$) are defined similarly.

Maximizing the log-likelihood function (eq. 5) with respect to the parameters will lead to a log-likelihood value for the given species tree, and the species tree that achieves the highest ℓ is the ML species tree. This is not analytically tractable. The program 3 s implements the method by explicitly summing over the gene trees (*G_i_*) and by using Gaussian quadrature to calculate the 2D integrals over $t_{i}$ (eq. 5) (Yang 2002; Zhu and Yang 2012; Dalquen et al. 2017). This is used in simulations.

We present two theorems for approximating the error in species tree estimation.

Theorem 1 .(a) *Suppose*  $z_{i}={\left(z_{i1},z_{i2},z_{i3}\right)}^{T},\;i=1,\ldots ,m$*, are an independent and identically distributed (i.i.d.) sample of size m from a distribution with means*  $\mu ={\left(\mu_{1},\mu_{2},\mu_{2}\right)}^{T}$*, with*  $\Delta \mu =\mu_{1}-\mu_{2}>0$*, and variances*  $\Sigma =\{\sigma_{jk}\}$*, where*  $\sigma_{11}=\sigma_{1}^{2},\;\sigma_{12}=\sigma_{13}=\rho_{12}\sigma_{1}\sigma_{2},\;\sigma_{22}=\sigma_{33}=\sigma_{2}^{2}$*and*  $\sigma_{23}=\rho_{23}\sigma_{2}^{2}$*. Let*  $\bar{z}={\left\{{\bar{z}}_{1},{\bar{z}}_{2},{\bar{z}}_{3}\right\}}^{T}$*be the sample means, with*  ${\bar{z}}_{j}=\frac{1}{m}\sum_{i=1}^{m}z_{ij},\;j=$*1, 2, 3. For large m*, $\bar{z}∼N_{3}(\mu ,\frac{1}{m}\Sigma )$*. Let*  $\zeta =P\{{\bar{z}}_{1}<\max({\bar{z}}_{2},{\bar{z}}_{3})\}$*. Then*

(6) $$\begin{array}{cc} \zeta & \approx \Phi \left(\frac{-\Delta \mu \sqrt{m}+\sqrt{\frac{1}{\pi }(\sigma_{2}^{2}-\sigma_{23})}}{\sqrt{\sigma_{1}^{2}-2\sigma_{12}+\sigma_{2}^{2}-\frac{1}{\pi }(\sigma_{2}^{2}-\sigma_{23})}}\right)\\ & \equiv \zeta_{N}(m,\Delta \mu ,\sigma_{1}^{2},\sigma_{2}^{2},\sigma_{12},\sigma_{23}), \end{array}$$

*where* Φ*is the cumulative distribution function (CDF) for the normal distribution*  N(0,1)*. We also write ζ_N_ as*  $\zeta_{N}(m,\Delta \mu ,\Sigma )$.

*(b) Let*  $a=s_{2}/s_{1}$*and*  $b=\Delta \mu \sqrt{m}/s_{1}$*, with*  $s_{1}^{2}=\sigma_{1}^{2}-2\sigma_{12}+\sigma_{2}^{2}-\frac{1}{2}(\sigma_{2}^{2}-\sigma_{23})$*and*  $s_{2}^{2}=\frac{1}{2}(\sigma_{2}^{2}-\sigma_{23})$*. Then ζ is bounded by:*

(7) $$\begin{array}{cc} \Phi (-h)(1+\frac{2}{\pi }{\;\text{tan}\;}^{-1}a) & \leq \zeta <2\Phi (-h)\\ & =\Phi (-h+\frac{1}{h}\text{log}\;2+o(\frac{1}{h})), \end{array}$$

*where*  $h=\frac{b}{\sqrt{1+a^{2}}}$*. The equality for the lower bound holds when*  h=b=0*. We write those bounds as*  $\zeta_{L1}\zeta \zeta_{U1}$*, so that*  $\Phi (-h)\leq \zeta_{L1}\leq \zeta <2\Phi (-h)$.

Proof. A proof is given in Appendix A, in which, we discuss alternative approximations and also give a tighter pair of bounds $\left(\zeta_{L2},\zeta_{U2}\right)$ in equation (A27), with $\zeta_{L1}<\zeta_{L2}<\zeta <\zeta_{U2}<\zeta_{U1}$. □

In this paper, *ζ* represents the error probability of species tree estimation. Thus, the bounds Φ(−h)≤ζ<2Φ(−h) suggest that when m→∞, the probit transform of the species-tree error probability, $\Phi^{-1}(\zeta )$, where $\Phi^{-1}$ is the inverse CDF of N(0,1), decreases linearly with $\sqrt{m}$. For practical calculations for finite *m* in this paper, equation (6) is more accurate (see Appendix A) and will be used later.

**Corollary 2***.Let (* $y_{0},y_{1},y_{2},y_{3}$*) be random variables from the multinomial distribution MN(m, q_0_, q_1_, q_2_, q_3_), with*  $q_{0}=1-q_{1}-q_{2}-q_{3},\;q_{1}>q_{2}$*= q_3_, and*  $\Delta q=q_{1}-q_{2}>0$*. Then*  $P\{y_{1}<\max(y_{2},y_{3})\}$*can be approximated by:*

(8) $$\zeta (m,q_{1},q_{2})=\Phi \left(\frac{-\Delta q\sqrt{m}+\sqrt{\frac{q_{2}}{\pi }}}{\sqrt{q_{1}+q_{2}-{\left(\Delta q\right)}^{2}-\frac{q_{2}}{\pi }}}\right),$$

(9) $$\zeta_{\text{ZLY}}(m,q_{1},q_{2})=\Phi \left(\frac{-\Delta q\sqrt{m-\frac{1}{\Delta q}}+\sqrt{\frac{q_{2}}{\pi }}}{\sqrt{q_{1}+q_{2}-\frac{q_{2}}{\pi }}}\right).$$

Proof. Let ${\bar{z}}_{j}=y_{j}/m,j=1,2,3$ be the observed frequencies. We have $\sigma_{jj}=q_{j}(1-q_{j})$ and $\sigma_{jk}=-q_{j}q_{k}$ for j≠k. Then equation (8) follows from equation (6) in Theorem 1. The form $\zeta_{\text{ZLY}}$, an alternative to equation (8), is from Yang (1996, eq. 3), based on Zharkikh and Li(1992, eq. 20). This applies the term 1/Δq to correct for discontinuity (Fleiss et al. 2003) and ignores correlations between *y*_1_, *y*_2_, and *y*_3_ as well as some terms of small probabilities. The discontinuity correction does not appear to be useful. If m≫1/Δq, both forms, with and without the discontinuity correction, are very close. □

The error rate for the ML method (eq. 5) is analyzed in Appendix B. When the number of loci m→∞, the MLE ${\hat{\theta }}_{j}\to \theta_{j}^{*}$ in species tree *S_j_*, *j *=* *1, 2, 3. Note that *S*_1_ represents the true model and $\theta_{1}^{*}$ are the true parameter values, while *S*_2_ and *S*_3_ are misspecified models and $\theta_{2}^{*}$ and $\theta_{3}^{*}$ are the “best-fitting or pseudotrue parameter values.” The Kullback–Leibler distance *D*_12_ from *S*_2_ to *S*_1_ is:

(10) $$\begin{array}{cc} D_{12}=\int f(x|S_{1},\theta_{1}^{*})\;\text{log}\;\frac{f(x|S_{1},\theta_{1}^{*})}{f(x|S_{2},\theta_{2}^{*})}\mathrm{dx}\\ & \;=E(l_{1}(\theta_{1}^{*}))-E(l_{2}(\theta_{2}^{*})), \end{array}$$

where $l_{j}(\theta_{j}^{*})\equiv \;\text{log}\;f(x|S_{j},\theta_{j}^{*})$, with *x* to be one data point (or site pattern counts at one locus), and where the integral means summation over all possible data outcomes at a locus. We use the per-locus log-likelihood values to compare the three species trees: ${\bar{z}}_{j}\equiv \frac{1}{m}\ell_{j}({\hat{\theta }}_{j})$, *j * == 1, 2, 3. When *m* is large, these have the means $E({\bar{z}}_{j})\approx E(l_{j}(\theta_{j}^{*}))\equiv \mu_{j}$, with $\mu_{1}-\mu_{2}=D_{12}$, and the variance matrix $\frac{1}{m}\Sigma$, where $\Sigma =\{\sigma_{jk}\}$ and $\sigma_{jk}\equiv \text{Cov}(l_{j}(\theta_{j}^{*}),l_{k}(\theta_{k}^{*}))$. The error of the ML method, $e_{ML}=P\{\ell_{1}({\hat{\theta }}_{1})<\max(\ell_{2}({\hat{\theta }}_{2}),\ell_{3}({\hat{\theta }}_{3}))\}$, is then given by Theorem 1 as:

(11) $$e_{ML}=P\{{\bar{z}}_{1}<\max({\bar{z}}_{2},{\bar{z}}_{3})\}\approx \zeta_{N}(m,D_{12},\Sigma ).$$

**Fig. 2.**
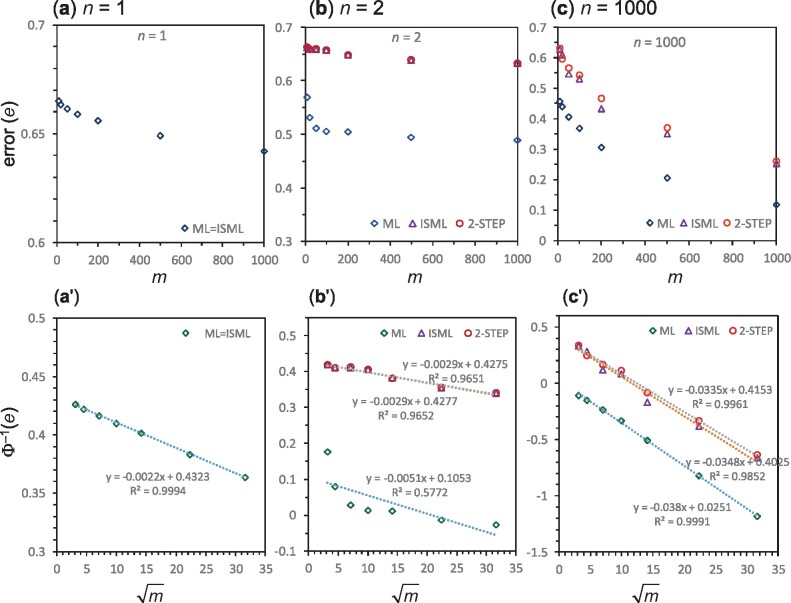
(*a–c*) Species-tree estimation error (*e*) at three sequence lengths (*n *=* *1, 2, 1,000) plotted against the number of loci (*m*) for different methods. (*a*′–*c*′) The probit transform of the species-tree error, $\Phi^{-1}(e)$, plotted against $\sqrt{m}$. The parameters used in the simulation are $\tau_{0}=0.02,\;\tau_{1}=0.019,\;\theta_{0}=0.01$, and $\theta_{1}=0.05$. When *n *=* *1, all four methods (ML, 2-step, concatenation, and isml) give the same species tree estimate, while concatenation and isml are equivalent in all cases considered in this paper. The number of replicates is $R\geq 10^{4}$ for ML and $\geq 10^{6}$ for the other methods.

Equation (11) cannot be used to calculate the error rate for ML as *D*_12_ and *σ_jk_* are not easily computable. It predicts a linear relationship between $\Phi^{-1}(e_{\text{ML}})$ and $\sqrt{m}$. This is confirmed by simulation (fig. 2*a*′–*c*′).

Precise results may be obtained in special cases. In the case of one locus (*m *=* *1), the ML gene tree is the ML species tree except for rare data sets: the true species tree *S*_1_ is recovered if $x_{i1}>\max(x_{i2},x_{i3})$. In rare data sets of extreme divergence, even if $x_{i1}>\max(x_{i2},x_{i3})$, ties for gene trees are possible, with the star tree being as good as the binary trees (Yang 2000), whereas ML under MSC favors *S*_1_. One such data set is $x_{i}=(4,13,12,11,50)$, in which case the three gene trees as well as the star tree achieve the same likelihood, whereas ML under MSC favors *S*_1_. However, such data sets involve sequences more divergent than random sequences have vanishingly small probability when *n* is large. Thus, we ignore them and consider all methods to be equivalent when *m *=* *1. With one locus, it is impossible to identify all parameters in the MSC model: there are four parameters and only three independent site-pattern frequencies ( $f_{i0},f_{i1},f_{i2}+f_{i3}$ for *S*_1_, for example).

The case of one site per locus (*n *=* *1) is analyzed later in the section on isml. Numerical calculations on a model species tree are presented in table 1. They will be discussed later in comparison with other methods.

In the case of n→∞, the gene tree (including the coalescent times) at each locus is given without errors. The likelihood is then the product of MSC densities of gene trees across the loci (eq. 2). This likelihood has singularities, with one or more species trees achieving infinite likelihood (Liu et al. 2010; Yang 2014). In the case of three species considered here, only one species tree (given by the smallest coalescent time) achieves infinite likelihood and will be the unique species-tree estimate, so that the estimation can proceed despite the singularity (Yang 2014, p. 360, Problem 9.4). Let the smallest coalescent/divergence time between species across all loci be *t_ab_*, *t_bc_*, and *t_ca_*. If *t_ab_* is the smallest among the three, then species tree *S*_1_ achieves infinite likelihood, by collapsing on the coalescent time *t_ab_*; that is, $\ell_{1}({\hat{\theta }}_{1})\to \infty$ as ${\hat{\tau }}_{0}={\hat{\tau }}_{1}=t_{ab}$ and ${\hat{\theta }}_{1}\to 0$ (see eq. 2) (Yang 2014, p.338–339), whereas the other two species trees have finite likelihood.

**Fig. 3.**
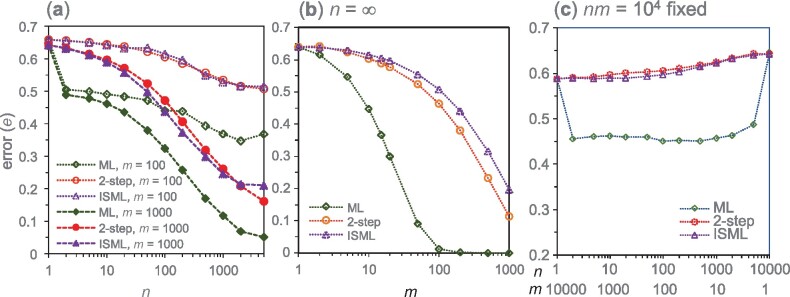
Error rates in species-tree estimation by ML, 2-step, and isml (=concatenation). (*a*) Error plotted against sequence length *n* when the number of loci *m* is fixed at 100 or 1,000, generated by simulation. (*b*) Error plotted against *m* when n=∞. Error for ML is given by equation (12), whereas those for isml and 2-step are generated by simulation. (*c*) Error plotted against *n* when $nm=10^{4}$ is fixed, generated by simulation. Note that all four methods are equivalent when *n *=* *1 or m=1, while concatenation and isml are equivalent in all cases. Parameters used in the simulation are $\tau_{0}=0.02,\;\tau_{1}=0.019,\;\theta_{0}=0.01$, and $\theta_{1}=0.05$. The number of replicates is $R\geq 10^{4}$.

Given *S*_1_ as the true species tree, both *t_bc_* and *t_ca_* are $>\tau_{\mathrm{ABC}}$ (fig. 1*b*). If sequences *a* and *b* coalesce in population *AB* at any of the *m* loci, *t_ab_* will be smaller than both *t_bc_* and *t_ca_*, and *S*_1_ will be the ML species tree. Thus, an incorrect species tree is inferred only if *a* and *b* do not coalesce in *AB* at any of the *m* loci and are not the first to coalesce in the root population *ABC*. Thus,

(12) $$e_{ML,\infty }=\phi^{m}\times \frac{2}{3},$$

where $\phi =e^{-\frac{2}{\theta_{AB}}(\tau_{ABC}-\tau_{AB})}$ is the probability that *a* and *b* do not coalesce in population *AB*. This equation is exact and applies to both small and large *m* (fig. 3*b*).

**Table 1.** Probabilities ($g_{1},g_{2},g_{3}$) of Estimated Gene Trees at Different Sequence Lengths (*n*) and the Error Rates for the Summary Methods 2-step and isml with *m *=* *1,000 Loci, Each with *n* Sites.

*n*	1	2	10	100	1,000	∞
2-step (mp-est)						
P(tie)	0.92948	0.8673	0.57015	0.22159	0.05105	0
$g_{1}(n)$	0.02378	0.04474	0.14515	0.26646	0.33273	0.35947
$g_{2}(n)=g_{3}(n)$	0.02337	0.04398	0.14235	0.25598	0.30811	0.32026
$e_{2-\mathrm{STEP}}$	0.642	0.633	0.597	0.470	0.260	0.114
$\zeta (m,g_{1},g_{2})$	0.644	0.635	0.600	0.472	0.264	0.113
$\zeta_{\text{ZLY}}(m,g_{1},g_{2})$	NA	NA	0.613	0.482	0.271	0.117
$\left(\zeta_{L1},\zeta_{U1}\right)$	(0.635, 0.953)	(0.623, 0.935)	(0.578, 0.869)	(0.430, 0.647)	(0.219, 0.331)	(0.087, 0.132)
$\left(\zeta_{L2},\zeta_{U2}\right)$	(0.637, 0.729)	(0.626, 0.714)	(0.585, 0.668)	(0.446, 0.561)	(0.242, 0.328)	(0.103, 0.132)
*ζ* (mean2)	0.683	0.670	0.627	0.504	0.285	0.118
*a*	0.574051	0.574056	0.573612	0.569708	0.562911	0.555962
*b*	0.0678913	0.0930368	0.190376	0.527747	1.11658	1.72268
isml (concat)						
$e_{\mathrm{ISML}}$	0.642	0.632	0.590	0.438	0.246	0.196
*ζ_N_*	0.644	0.634	0.592	0.443	0.254	0.194
$\zeta_{N0}$	0.643	0.633	0.591	0.437	0.234	0.166
$\left(\zeta_{L1},\zeta_{U1}\right)$	(0.635, 0.953)	(0.622, 0.934)	(0.568, 0.854)	(0.397, 0.598)	(0.211, 0.318)	(0.157, 0.237)
$\left(\zeta_{L2},\zeta_{U2}\right)$	(0.637, 0.728)	(0.625, 0.713)	(0.576, 0.659)	(0.416, 0.536)	(0.233, 0.316)	(0.177, 0.237)
*ζ* (mean2)	0.683	0.669	0.618	0.476	0.275	0.207
*a*	0.574029	0.573971	0.57356	0.569747	0.558232	0.553151
*b*	0.067892	0.0958963	0.21228	0.607057	1.14253	1.35035

Note.—P(tie) is the probability for ties in gene trees, with $P(\text{tie})+g_{1}+2g_{2}=$ 1. The probabilities of estimated gene trees ($g_{1},g_{2},g_{3}$) as well as the error rates ($e_{2-\mathrm{STEP}}$ and $e_{\text{ISML}}$) are estimated by simulation using a C program, with $\geq 10^{6}$ replicates. Ties are broken evenly in the error calculation. The parameter values used are $\left(\tau_{0},\tau_{1},\theta_{0},\theta_{1}\right)$=(0.02, 0.019, 0.01, 0.05). The marginal (pooled) site pattern probabilities are $\bar{p}=$ (${\bar{p}}_{0},\;{\bar{p}}_{1},\;{\bar{p}}_{2},\;{\bar{p}}_{3},\;{\bar{p}}_{4}$) = (0.92831926, 0.023777106, 0.023372801, 0.023372801, 0.001158033), given by equation (13). For 2-step, at *n *=* *1, the estimated gene tree is determined by the single site so that $g_{1}(1)={\bar{p}}_{1}$and $g_{2}(1)={\bar{p}}_{2}$, whereas at n=∞, the estimated gene tree is the true gene tree, so that $g_{1}(\infty )=P(G_{1})$ and $g_{2}(\infty )=P(G_{2})$ (eq. 1). For 2-step, $\zeta_{\text{ZLY}}$ (eq. 9) is inapplicable at *n *=* *1 or 2 as *m *=* *1000 is too small. For isml, $\zeta_{N0}=\zeta_{N}(m,\Delta \mu ,\sigma_{1}^{2},\sigma_{2}^{2},0,0)$ ignores the correlation (eq. 6), while *ζ_N_* accounts for the correlation. The bounds $\left(\zeta_{L1},\zeta_{U1}\right)$ and $\left(\zeta_{L2},\zeta_{U2}\right)$ are calculated using equations (7) and (A27), with *k *=* *2 used in $\zeta_{U2}$. “mean2” is the average of the tight bounds: $(\zeta_{L2}+\zeta_{U2})/2$.

### Concatenation

Sequence alignments at the *m* loci are merged into a super-alignment of length *nm*, and the data are the site-pattern counts pooled across loci: $x.=\{x_{\cdot j}\}$, with $x_{\cdot j}=\sum_{i}x_{ij},\;j=0,1,\ldots ,4$. The likelihood function is given by the multinomial probability of equation (4) except that $x_{\cdot j}$ is used instead of *x_ij_*. The ML tree is *G*_1_ if $x_{\cdot 1}>\max(x_{\cdot 2},x_{\cdot 3})$ (Yang 1994b, 2000). We discuss the error rate of concatenation below in the section on the isml method.

We also examine biases in parameter estimation using concatenation. We use species tree *S*_1_ with *τ_ABC_* = 0.02, *τ_AB_* = 0.01, *θ_ABC_* = 0.02, and *θ_AB_* = 0.01 to simulate $m=10^{4}$ loci each with *n *=* *250 sites. We obtain MLEs ${\hat{t}}_{0}$ and ${\hat{t}}_{1}$ on gene tree *G*_1_ from the concatenated data for comparison with the MLEs ${\hat{\tau }}_{0}$ and ${\hat{\tau }}_{1}$ on species tree *S*_1_ in the MSC model (eq. 5). With so much data, both concatenation and ML recover the true tree with near certainty. The MLEs under the MSC (obtained using the 3 sprogram) are very close to the true values, whereas concatenation (baseml in paml, Yang 2007) produced seriously biased estimates (table 2). Even the relative age, ${\hat{t}}_{0}/{\hat{t}}_{1}$ = 1.92, differs from $\tau_{\mathrm{ABC}}/\tau_{AB}=2$, which means that molecular clock dating analysis using concatenated data will produce biased time estimates (Angelis and dos Reis 2015; Ogilvie et al. 2017; Tiley et al. 2020).

**Table 2.** Estimates of Divergence Times (true values in parentheses) by ML under the MSC (3 s) and by Concatenation (baseml) in Two Simulated Data Sets, Each of $m=10^{4}$ Loci and *n *=* *250 Sites.

	*τ_ABC_*	*τ_AB_*	*θ_ABC_*	*θ_AB_*
Data/method	(0.02)	(0.01)	(0.02)	(0.01)
Data set 1, 3s	0.0201	0.0096	0.0199	0.0101
Data set 2, 3s	0.0196	0.0100	0.0201	0.0100
Data set 1, baseml	0.0298	0.0155		
Data set 2, baseml	0.0298	0.0156		

### ISML

The isml method assumes that all sites in the super-alignment are i.i.d. Like concatenation, the data are summarized as pooled site-pattern counts, $x.=\{x_{\cdot 0},x_{\cdot 1},x_{\cdot 2},x_{\cdot 3},x_{\cdot 4}\}$. However, isml is coalescent-aware and uses the MSC model to calculate the probabilities for the site patterns. By averaging the conditional site-pattern probabilities of equation (3) over the MSC density of gene trees and coalescent times of equation (2), we derive the marginal site-pattern probabilities, $\bar{p}=({\bar{p}}_{0},\ldots ,{\bar{p}}_{4})$, as:

(13) $$\begin{array}{c} {\bar{p}}_{0}=\frac{1}{16}\left(1+18a_{0}+54a_{0}b+54a_{0}c_{0}+9c_{1}+9a_{1}\right),\\ {\bar{p}}_{1}=\frac{3}{16}\left(1-6a_{0}-18a_{0}b-18a_{0}c_{0}+9c_{1}+9a_{1}\right),\\ {\bar{p}}_{2}=\frac{3}{16}\left(1+6a_{0}-18a_{0}b-18a_{0}c_{0}-3c_{1}-3a_{1}\right),\\ {\bar{p}}_{3}={\bar{p}}_{2},\\ {\bar{p}}_{4}=\frac{6}{16}\left(1-6a_{0}+18a_{0}b+18a_{0}c_{0}-3c_{1}-3a_{1}\right), \end{array}$$

where $a_{0}=\frac{e^{-8\tau_{0}/3}}{3+4\theta_{0}}$, $a_{1}=\frac{e^{-8\tau_{1}/3}}{3+4\theta_{1}}$, $b=\frac{e^{-4\tau_{1}/3}}{3+2\theta_{1}}$, $c_{0}=2\phi \cdot (\theta_{1}-\theta_{0})\cdot \frac{e^{-4\tau_{0}/3}}{\left(3+2\theta_{0})(3+2\theta_{1}\right)}$, and $c_{1}=4\phi \cdot (\theta_{1}-\theta_{0})\cdot \frac{a_{0}}{3+4\theta_{1}}$, with $\phi =e^{-2(\tau_{0}-\tau_{1})/\theta_{1}}$. Note that $\left\{{\bar{p}}_{j}\right\}$ are functions of *a*_0_, $b+c_{0}$ and $a_{1}+c_{1}$, although these do not appear to permit simple biological interpretations. The cases for *S*_2_ and *S*_3_ are given by symmetry.

The likelihood function (or the probability for the pooled site-pattern counts) for each species tree is:

(14) $$\begin{array}{c} f(x.|S_{1},\theta_{1})={\bar{p}}_{0}^{x_{\cdot 0}}{\bar{p}}_{1}^{x_{\cdot 1}}{\bar{p}}_{2}^{x_{\cdot 2}+x_{\cdot 3}}{\bar{p}}_{4}^{x_{\cdot 4}},\\ f(x.|S_{2},\theta_{2})={\bar{p}}_{0}^{x_{\cdot 0}}{\bar{p}}_{1}^{x_{\cdot 2}}{\bar{p}}_{2}^{x_{\cdot 3}+x_{\cdot 1}}{\bar{p}}_{4}^{x_{\cdot 4}},\\ f(x.|S_{3},\theta_{3})={\bar{p}}_{0}^{x_{\cdot 0}}{\bar{p}}_{1}^{x_{\cdot 3}}{\bar{p}}_{2}^{x_{\cdot 1}+x_{\cdot 2}}{\bar{p}}_{4}^{x_{\cdot 4}}. \end{array}$$

Theorem 3 *.(a) If the true species tree is S_1_ with parameters*  $\theta_{1}$*, then*  ${\bar{p}}_{1}>{\bar{p}}_{2}={\bar{p}}_{3}$*. (b) Isml infers the species tree S_1_ if*  $x_{\cdot 1}>\max\{x_{\cdot 2},x_{\cdot 3}\}$.

Proof. (a) Each of the marginal site pattern probabilities ${\bar{p}}_{j},\;j=0,\ldots ,4$, is a sum over the four gene trees of figure 1*b*:$G_{1a},G_{1b},G_{2}$ and *G*_3_. The three gene trees $G_{1b},G_{2}$, and *G*_3_ have the same densities (eq. 2). Together their contribution to the site pattern *xxy* is the same as that to the pattern *yxx* or pattern *xyx*. If the gene tree is $G_{1a}$ (with any coalescent times *t*_0_ >* t*_1_), site pattern *xxy* will have a higher probability than *yxx* or *xyx*, with $p_{1}>p_{2}=p_{3}$. Averaging over all the four gene trees, we have ${\bar{p}}_{1}>{\bar{p}}_{2}={\bar{p}}_{3}$.

(b) We show that if $x_{\cdot 1}>x_{\cdot 2}$, then $\ell (S_{1},{\hat{\theta }}_{1})>\ell (S_{2},{\hat{\theta }}_{2})$, where ${\hat{\theta }}_{1}$ and ${\hat{\theta }}_{2}$ are the MLEs under each species tree. First note that if $x_{\cdot 1}>x_{\cdot 2}$ and $q_{1}>q_{2}>0$, then $q_{1}^{x_{\cdot 1}}q_{2}^{x_{\cdot 2}}>q_{1}^{x_{\cdot 2}}q_{2}^{x_{\cdot 1}}$. Let $q_{1}={\bar{p}}_{1}(S_{1},{\hat{\theta }}_{2})$and $q_{2}={\bar{p}}_{2}(S_{1},{\hat{\theta }}_{2})$, and we have $\ell (S_{1},{\hat{\theta }}_{2})>\ell (S_{2},{\hat{\theta }}_{2})$. In other words, even if we use ${\hat{\theta }}_{2}$ (the MLE for *S*_2_) to calculate the likelihood for species tree *S*_1_, tree *S*_1_ will have a higher likelihood than *S*_2_. Since ${\hat{\theta }}_{2}$ may not be optimal for *S*_1_, it follows that $\ell (S_{1},{\hat{\theta }}_{1})\geq \ell (S_{1},{\hat{\theta }}_{2})>\ell (S_{2},{\hat{\theta }}_{2})$. □

Theorem 3 means that isml infers species tree *S_j_* if $x_{\cdot j}$ is the greatest among $x_{\cdot 1},\;x_{\cdot 2}$, and $x_{\cdot 3}$, just like concatenation.

To study the error rate for isml (or concat), let *p_ij_*, j=0,…,4 be the site-pattern probabilities at any locus *i*. Data at each locus are represented by the site-pattern frequencies $f_{ij}=x_{ij}/n$. Let $f_{i}=\{f_{ij}\}$ be the data at locus *i*. The *f_i_* are i.i.d. among loci from a common distribution with mean $E(f_{ij})={\bar{p}}_{j}$ and variance/covariance $\sigma_{jj}\equiv V(f_{ij})$ and $\sigma_{jk}\equiv \text{Cov}(f_{ij},f_{ik})$. Let ${\bar{f}}_{j}=\frac{1}{m}\sum_{i=1}^{m}f_{ij}=x_{\cdot j}/m$ be the means over loci. Here, $\left\{f_{ij}\right\}$ constitute the full data, whereas $\left\{{\bar{f}}_{j}\right\}$ are summaries used by isml: the species tree estimate is *S_j_* if ${\bar{f}}_{j}$ is the largest among $\left({\bar{f}}_{1},{\bar{f}}_{2},{\bar{f}}_{3}\right)$. Thus, $e_{\mathrm{ISML}}=P({\bar{f}}_{1}<\max\{{\bar{f}}_{2},{\bar{f}}_{3}\})\approx \zeta_{N}(m,{\bar{p}}_{1}-{\bar{p}}_{2},\Sigma )$, where $\Sigma =\{\sigma_{jk}\}$. Below we derive the variances.

At *n *=* *1, they are given by the multinomial distribution as:

(15) $$\sigma_{jj}^{\left(1\right)}={\bar{p}}_{j}(1-{\bar{p}}_{j}),\;\sigma_{jk}^{\left(1\right)}=-{\bar{p}}_{j}{\bar{p}}_{k},\;1\leq j,k\leq 3.$$

At n=∞, we have *f_ij_* =* p_ij_*, given by equation (3). The variances, denoted $\sigma_{jk}^{\left(\infty \right)}$, can be generated by simulating gene trees with coalescent times and calculating the site-pattern probabilities (eq. 3) (supplementary table S1, Supplementary Material online). This distribution is 3D (for $f_{i0}$, $f_{i1}$, and $f_{i2}=f_{i3}$ under *S*_1_), indexed by four parameters ($\theta_{1}$ in *S*_1_), and is a mixture distribution with 4 components corresponding to the four gene trees of figure 1*b*. It reflects the coalescent fluctuation in gene genealogies.

For any finite 1≤n<∞, the variances are given by:

(16) $$\begin{array}{c} \sigma_{jj}=V(E(f_{ij}|p_{ij}))+E(V(f_{ij}|p_{ij}))\\ =V(p_{ij})+E(p_{ij}(1-p_{ij})/n)\\ =V(p_{ij})+\frac{1}{n}[E(p_{ij})(1-E(p_{ij}))-V(p_{ij})]\\ =\frac{1}{n}\sigma_{jj}^{\left(1\right)}+\frac{n-1}{n}\sigma_{jj}^{\left(\infty \right)},\\ \sigma_{jk}=\text{Cov}(p_{ij},p_{ik})+E(\text{Cov}(f_{ij},f_{ik}|p_{ij},p_{ik}))\\ =\text{Cov}(p_{ij},p_{ik})+\frac{1}{n}[-E(p_{ij})E(p_{ik})-\text{Cov}(p_{ij},p_{ik})]\\ =\frac{1}{n}\sigma_{jk}^{\left(1\right)}+\frac{n-1}{n}\sigma_{jk}^{\left(\infty \right)}, \end{array}$$

where $E(p_{ij})\equiv {\bar{p}}_{j}$ (eq. 13), whereas $V(p_{ij})=\sigma_{jj}^{\infty }$ and $\text{Cov}(p_{ij},p_{ik})=\sigma_{jk}^{\infty }$ are the variances/covariances over the coalescent process. These are calculated for a set of parameter values in supplementary table S1, Supplementary Material online. The variances of *f_ij_* are thus weighted averages of variances at n=1 and ∞.

**Fig. 4.**
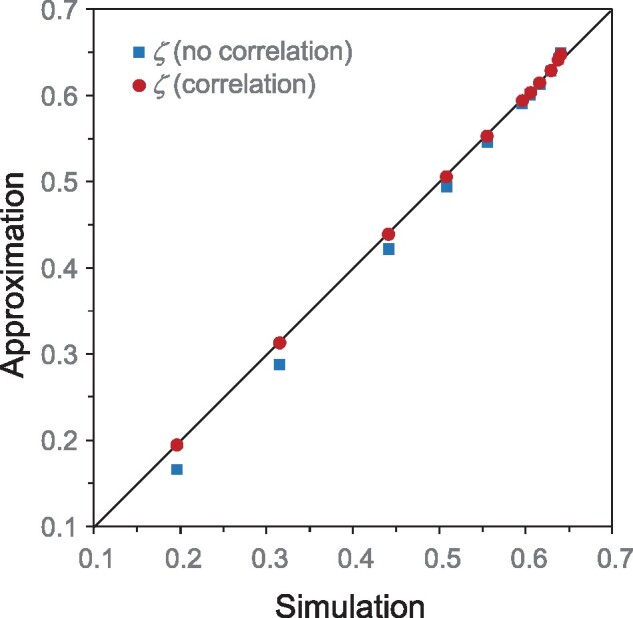
Species tree error for isml at n=∞ generated by simulation (10^8^ replicates) and by approximation based on *ζ_N _*either with or without accounting for correlations. The error goes from 0.64 (at *m *=* *1) to 0.19 (at *m *=* *1,000). Results for other methods for the same parameter settings are in figure 3*b*.

The approximation $e_{\mathrm{ISML}}\approx \zeta_{N}(m,{\bar{p}}_{1}-{\bar{p}}_{2},\Sigma )$ is very accurate, with errors <0.002 in the simulation of table 1. At large *n*, accommodating correlation is useful as $\zeta_{N0}$ which ignores correlation is less accurate (see fig. 4 for the case of n=∞). For example, the correlation $\rho (f_{i1},f_{i2})=\frac{\sigma_{12}}{\sqrt{\sigma_{11}\sigma_{22}}}$is −0.124,−0.153, and −0.181 at *n *=* *1, 1,000, and ∞, respectively (supplementary table S1, Supplementary Material online).

We now consider parameter estimation by isml. Theorem 3 allows species tree estimation by isml without knowledge of the MLE of the parameters. With data of $x._{j},j=0,\ldots ,4$, there are only three observations (three free proportions ${\bar{f}}_{0},\;{\bar{f}}_{1}$, and ${\bar{f}}_{2}+{\bar{f}}_{3}$ in the case of *S*_1_). As there are four parameters in the MSC model, it is impossible to identify all of them.

If we assume $\theta_{0}=\theta_{1}=\theta$ (as in Tian and Kubatko 2016), all three parameters ($\tau_{0},\tau_{1},\theta$) will be identifiable. As $c_{0}=c_{1}=0$, equation (13) simplifies to:

(17) $$\begin{array}{cc} {\bar{p}}_{0} & =\frac{1}{16}\left(1+18a_{0}+54a_{0}b+9a_{1}\right),\\ {\bar{p}}_{1} & =\frac{3}{16}\left(1-6a_{0}-18a_{0}b+9a_{1}\right),\\ {\bar{p}}_{2} & =\frac{3}{16}\left(1+6a_{0}-18a_{0}b-3a_{1}\right)={\bar{p}}_{3},\\ {\bar{p}}_{4} & =\frac{6}{16}\left(1-6a_{0}+18a_{0}b-3a_{1}\right), \end{array}$$

where *a*_0_, *a*_1_, and *b* are defined in equation (13) with $\theta_{0}=\theta_{1}=\theta$. By equating the observed site-pattern frequencies to their expected probabilities (eq. 17), we have

(18) $$\begin{array}{c} \frac{1}{4}(9a_{1}+1)={\bar{f}}_{0}+{\bar{f}}_{1}\equiv h_{1},\\ \frac{1}{4}(9a_{0}+1)={\bar{f}}_{0}+\frac{1}{2}({\bar{f}}_{2}+{\bar{f}}_{3})\equiv h_{2},\\ \frac{3}{8}(-18a_{0}b+3a_{1}+1)={\bar{f}}_{1}+\frac{1}{2}({\bar{f}}_{2}+{\bar{f}}_{3})\equiv h_{3}. \end{array}$$

Thus, we have a quadratic equation in $\hat{\theta }$:

(19) $$\begin{array}{c} 4{\left(4h_{3}-2h_{1}-1\right)}^{2}{\hat{\theta }}^{2}+[3{\left(4h_{3}-2h_{1}-1\right)}^{2}\\ -(4h_{1}-1){\left(4h_{2}-1\right)}^{2}](4\hat{\theta }+3)=0. \end{array}$$

This always has a unique positive root. Given $\hat{\theta }$, the estimates ${\hat{\tau }}_{0}$ and ${\hat{\tau }}_{1}$ are given by equation (18), which are guaranteed to be positive.

Thus, under the assumption $\theta_{0}=\theta_{1}$, the isml method provides estimates of the three parameters in the model: *θ*, *τ*_0_, and *τ*_1_. As there is a one-to-one correspondence between the parameters and the multinomial proportions, the estimates are consistent and approach the true values when m→∞ for any n≥1 if the assumption of $\theta_{0}=\theta_{1}$ is correct (table 3, cases c and d). However, the pooled site-pattern counts or average site-pattern frequencies are summaries of the original data and are not sufficient statistics. It then follows that the isml estimates will be less efficient and have larger asymptotic variances than the MLEs obtained from the full data under the same model assumption of $\theta_{0}=\theta_{1}$ (table 3, case c). Furthermore, if $\theta_{0}\neq \theta_{1}$, assuming $\theta_{0}=\theta_{1}$ will lead to biased and inconsistent parameter estimates even if the same species tree estimate is produced. In other words if $\theta_{0}\neq \theta_{1}$, the isml method assuming $\theta_{0}=\theta_{1}$ will produce a consistent estimate of the species tree and inconsistent estimates of the model parameters (table 3, cases e and f).

**Table 3.** Characterization of the isml Method.

	True Model	Assumption	Data Size	Parameters	isml vs. ml
(a)	$\theta_{0}\neq \theta_{1}$	$\theta_{0}\neq \theta_{1}$	*n *>* *1	3 out of 4 identifiable	isml ≠ ml
(b)	$\theta_{0}\neq \theta_{1}$	$\theta_{0}\neq \theta_{1}$	*n *=* *1	3 out of 4 identifiable	isml = ml
(c)	$\theta_{0}=\theta_{1}$	$\theta_{0}=\theta_{1}$	*n *>* *1	all 3 identifiable	isml ≠ ml
(d)	$\theta_{0}=\theta_{1}$	$\theta_{0}=\theta_{1}$	*n *=* *1	all 3 identifiable	isml = ml
(e)	$\theta_{0}\neq \theta_{1}$	$\theta_{0}=\theta_{1}$	*n *>* *1	3 out of 4 identifiable, inconsistent	isml ≠ ml
(f)	$\theta_{0}\neq \theta_{1}$	$\theta_{0}=\theta_{1}$	*n *=* *1	3 out of 4 identifiable, inconsistent	isml = ml

Note.—In all cases, the species tree topology is identifiable and consistently estimated by isml when the number of loci m→∞. If the parameters are identifiable, their estimates will be consistent. When isml differs from ML and the assumed model is correct, isml is less efficient than ML for parameter estimation (case c).

### Two-Step Method (Majority Vote)

In the 2-step method, we estimate gene trees at individual loci and then use the most common gene tree topology as the species tree estimate. Under JC, the ML gene tree for locus *i* (which is also the upgma tree) is tree *G_j_* if *x_ij_* is the largest among $x_{i1},x_{i2}$, and $x_{i3}$ (Yang 1994b, 2000); site patterns *xxy*, *yxx*, and *xyx* “support” gene trees *G*_1_, *G*_2_, and *G*_3_, respectively. There is no need for numerical optimization to obtain the ML tree at each locus.

Let *g*_1_, *g*_2_, and *g*_3_ be the probabilities that the estimated gene tree is *G*_1_, *G*_2_, and *G*_3_, respectively; that is, $g_{1}=P\{x_{i1}>\max(x_{i2},x_{i3})\}$, and so on. These are functions of all four parameters in the MSC model ($\theta_{1}$) as well as the sequence length *n*, and can be computed numerically (Yang 2002, eq. 12) or by simulation. Under JC and the clock, $g_{2}=g_{3}<g_{1}<P(G_{1}|S_{1},\theta_{1})$ (Yang 2002). This result has several implications. First, $g_{1}<P(G_{1})$ means that phylogenetic errors inflate gene-tree–species-tree discordance and lead to underestimation of the internal branch length in the species tree (Yang 2002). Second $g_{1}<P(G_{1})$ also means that use of estimated (rather than true) gene trees leads to reduced probability for recovering the correct species tree. Third, $g_{1}>g_{2}=g_{3}$ means that the 2-step estimate of the species tree is consistent even if estimated gene trees are used.

Let the number of loci at which *G*_1_ is the ML tree be $m_{1}=\sum_{i=1}^{m}I_{x_{i1}>\max(x_{i2},\;x_{i3})}$, where the indicator function $I_{a}=1$ if statement *a* is true and 0 otherwise. Similarly define *m*_2_ and *m*_3_ to be the counts for the two mismatching gene trees. The correct species tree is inferred if and only if $m_{1}>\max(m_{2},m_{3})$. Thus, the error rate can be approximated by $e_{2-\mathrm{STEP}}\approx \zeta (m,g_{1},g_{2})$ (eq. 8).

The accuracy of this approximation is assessed in table 1 at different values of *n* with *m *=* *1,000 and with parameter values $\tau_{0}=0.02,\;\tau_{1}=0.019,\;\theta_{0}=0.01$, and $\theta_{1}=0.05$. Consider first the case of *n *=* *1. The gene tree is resolved if the single site at the locus has site patterns 1, 2, or 3, but is unresolved if the site has patterns 0 or 4. Whether we ignore loci with ties (with site patterns 0 or 4) or break ties evenly (assigning $\frac{1}{3}$ to each gene tree) does not affect the species tree estimate. Thus, $g_{1}(1)={\bar{p}}_{1}$ and $g_{2}(1)={\bar{p}}_{2}$ (eq.13) and the error is $e_{2-\mathrm{STEP}}\approx \zeta (m,{\bar{p}}_{1},{\bar{p}}_{2})$. This is equivalent to $e_{\mathrm{ISML}}\approx \zeta_{N}(m,{\bar{p}}_{1}-{\bar{p}}_{2},\Sigma )$ for isml, consistent with the fact that at *n *=* *1 all methods considered here are equivalent.

If n=∞, the estimated gene trees will be the true gene trees so that $g_{1}=P(G_{1})$ and $g_{2}=P(G_{2})$. The error rate is then $\zeta (m,P(G_{1}),P(G_{2}))=$*ζ*(1,000, 0.3594737, 0.3202631) = 0.1132, close to 0.114 from simulation. At n=1000, the proportions of estimated gene trees are *g*_1_ = 0.33273 and *g*_2_ = 0.30811, so that $\zeta (m,g_{1},g_{2})=$ 0.264, close to 0.260 by simulation (table 1). These are much larger than 0.114 at n=∞, suggesting that with *n *=* *1,000 sites in the sequence, the estimated gene trees have substantial errors and uncertainties.

The approximations $\zeta_{\text{ZLY}}$ (eq. 9) and *ζ* (eq. 8) give nearly identical results. The error rate is found to be very sensitive to the precise values of *g*_1_ and *g*_2_. Overall, the approximation is good, with errors within or close to 1%.

### Numerical Comparison of Different Methods

We use simulation to compare the different species-tree estimation methods and to assess the reliability of our approximations. We use a challenging species tree with parameters $\tau_{0}=0.02,\;\tau_{1}=0.019,\;\theta_{0}=0.01$, and $\theta_{1}=0.05$. The error is plotted against the number of loci (*m*) when the number of sites per locus is fixed at n= 1, 2, or 1,000 (fig. 2).

In the case of one site per sequence (*n *=* *1), all four methods considered in this study are equivalent, with the species tree given by the most frequent pooled site pattern (i.e., the greatest of $x_{\cdot 1},\;x_{\cdot 2}$, and $x_{\cdot 3}$). With one site, the independent-sites assumption is correct, and ml and isml are exactly the same. As discussed earlier, concatenation and 2-step also select the species tree according to the pooled site patterns. Treatment of ties among $x_{\cdot 1},x_{\cdot 2},x_{\cdot 3}$ has very minor effects on the error rate. For *n *=* *1 and m=1000, simulation gave the error estimate e= 0.642 if ties are broken evenly (table 1) or 0.641 if data sets with ties are ignored. As predicted by our theory, the probit transform of the error, $\Phi^{-1}(e)$, shows a linear relationship with $\sqrt{m}$ (fig. 2*a′*, $R^{2}=0.9994$).

In the case of *n *=* *2 sites per locus, isml (=concatenation), 2-step, and ML are all distinct. To see that concatenation and 2-step may produce different species trees, consider the case of *m *=* *3 loci and *n *=* *2 sites. If the data set at the three loci are 11, 02, and 00, where 0–4 represent the five site patterns, concatenation will infer the correct species tree *S*_1_ (as $x_{\cdot 1}=2,x_{\cdot 2}=1,x_{\cdot 3}=0$), whereas 2-step will have a tie between *S*_1_ and *S*_2_ (as $m_{1}=1,m_{2}=1,m_{3}=0$). If the data set at the three loci are 33, 01, and 14, concatenation will have a tie between *S*_1_ and *S*_3_ (as $x_{\cdot 1}=2,x_{\cdot 2}=0,x_{\cdot 3}=2$), whereas 2-step will infer the correct species tree (as $m_{1}=2,m_{2}=0,m_{3}=1$). We also confirm that at *n *=* *2 ML differs from all three summary methods and can identify and consistently estimate all four parameters in the MSC model. Indeed ML is far more efficient for species tree estimation than the summary methods when *n *=* *2 (fig. 2*b* and *b*′). Although the summary methods improve only slightly when *n* changes from 1 to 2, there is a major performance boost for ML (fig. 3*a*). This may be due to the fact that the model is fully identifiable with *n *=* *2 but not when *n *=* *1. The predicted linear relationship between $\Phi^{-1}(e)$ and $\sqrt{m}$ holds well for the three summary methods (fig. 2*b*′). For ML, if we remove the first two points (for *m *=* *10 and 20), the relationship is nearly linear, with y=−0.0022x+0.0391, with $R^{2}=0.97$.

The most interesting case is with n≫1, since in real data sets *n* may be in the range 50–5,000, say. We used *n *=* *1,000 in figure 2*c* and *c*′. As in the case of *n *=* *2, there is a large performance divide between ML and the three summary methods (Isml = concat and 2-step), whereas the summary methods have similar performance. The approximate linear relationship between $\Phi^{-1}(e)$ and $\sqrt{m}$ holds well for all methods.

The superior performance of ML persists in the limit of n=∞ (fig. 3*b*). For example, $e_{\text{ML},\infty }=$ 0.45 and 0.01 for ML at *m *=* *10 and 100, respectively, compared with $e_{2-\mathrm{STEP},\infty }=$ 0.60 and 0.46 for 2-step or $e_{\mathrm{ISML},\infty }=$ 0.62 and 0.51 for isml. The differences between ML and 2-step reflect the information in the coalescent times or gene-tree branch lengths. The differences between ML and isml reflect the information in the variation of site-pattern frequencies among loci, as isml uses only the averages across loci.

Figure 3*c* examines the error rates of different methods, while $nm=10^{4}$ is fixed. At the two ends (*n *=* *1 or *m *=* *1), all four methods are equivalent, with e= 0.587 at *n *=* *1 and $m=10^{4}$, and e= 0.646 at *m *=* *1 and $n=10^{4}$. Note that when n=1 and m→∞, the error e→0, while if *m *=* *1 and n→∞, the error $e=1-g_{1}(n)\to 1-P(G_{1})=$ 0.6405. The high error at *m *=* *1 even when n=∞ is because a single gene tree (with coalescent times), even if known with certainty, does not contain much information about the MSC process. Away from the two ends (*n *>* *1 or *m *>* *1), ML is considerably more efficient than the summary methods (fig. 3*c*). The case of $m=10^{4}$ (*n *=* *1), at which $e_{\text{ML}}=$ 0.587, and the case of *m *=* *2 (*n *=* *5,000), at which $e_{\text{ML}}$ = 0.487, make an interesting contrast. In the first case all sites are i.i.d., while in the second, there are only two independent genes, each of 5,000 sites in complete linkage. One might expect data of independent sites to be more informative than two loci with correlated sites at the same locus (e.g., Long and Kubatko 2018), but the opposite is true. With *n *=* *1, not all model parameters are identifiable, and this nonidentifiability issue appears to impact species tree estimation as well (Shi and Yang 2018, p. 172). With *nm* fixed, the smallest error $e_{\text{ML}}$ occurs at intermediate values of *n* and *m*, around n=m=100, although performance is similar over a large range of *n* (fig. 3*c*).

In table 1, we calculated the species-tree error probability using equations (6) and (8), as well as two pairs of bounds ($\zeta_{L1},\zeta_{U1}$) and ($\zeta_{L2},\zeta_{U2}$) (Theorem 1, Appendix A), for comparison with the simulation results. The asymptotic results are expected to apply when the sequence length *n* is fixed, whereas the number of loci m→∞. Here, *m* is fixed at 1,000, so that *b *<* *2 for all cases (table 1), and is too small for the asymptotic approximations to be reliable. As a result, equations (6) and (8) are more accurate.

## Discussion

### Errors of Species Tree Estimation by Different Methods

Under the MSC model, data at different loci are i.i.d., so that the number of loci (*m*) constitutes the sample size in the statistical model. Thus, we have derived approximations to the error rate for different methods when *m* increases, with the sequence length *n* fixed. For large *m*, the error can be approximated by $\Phi (-c\sqrt{m})$, where *c* is a constant. This is seen to apply to all four methods considered in this study (ML, isml = concatenation, and 2-step) (see table 4 for a summary).

**Table 4.** Summary of Analytical Approximations to Species-Tree Estimation Error by Different Methods.

Method	*n *=* *1	n≥2	n=∞
ml		eq. 11	eq. 12
2-step	$\zeta (m,{\bar{p}}_{1},{\bar{p}}_{2})$	$\zeta (m,g_{1},g_{2})$	$\zeta (m,P(G_{1}),P(G_{2}))$
isml/concatenation	$\zeta_{N}(m,\Delta p,\Sigma^{\left(1\right)})$	$\zeta_{N}(m,\Delta p,\Sigma^{\left(n\right)})$	$\zeta_{N}(m,\Delta p,\Sigma^{\left(\infty \right)})$

Note.—For isml/concatenation, $\Delta p={\bar{p}}_{1}-{\bar{p}}_{2}$, and the variance–covariance matrix at *n* is $\Sigma^{\left(n\right)}=\frac{1}{n}\Sigma^{\left(1\right)}+\frac{n-1}{n}\Sigma^{\left(\infty \right)}$ (eq. 16). In the case of *n *=* *1, $\zeta (m,{\bar{p}}_{1},{\bar{p}}_{2})=\zeta_{N}(m,\Delta p,\Sigma^{\left(1\right)})$, and 2-step, isml, concatenation, and ml are all equivalent.

**Fig. 5.**
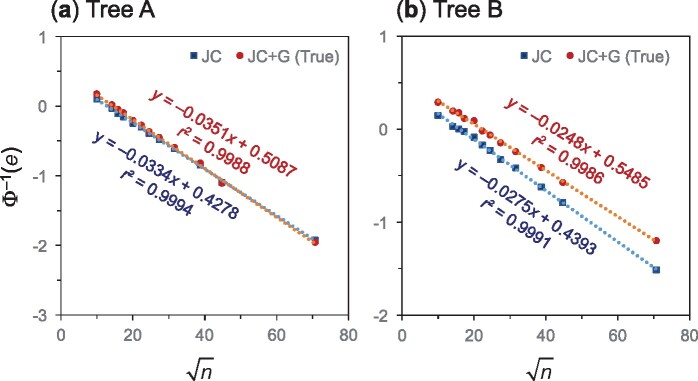
The probit transform of the phylogenetic reconstruction error, $\Phi^{-1}(e)$, is a linear function of the square root of the number of sites in the alignment ($\sqrt{n}$). Simulation results from Yang (1997, fig. 1A and B) are used in the plot. The trees used in the simulation have four taxa, with branch lengths ((0.5, 0.5):0.1, 0.5, 0.5) for tree A and ((0.5, 0.5):0.1, 0.6, 1.4) for tree B. Data are simulated under the JC+G model (Yang 1994a) and analyzed under both JC and JC+G (Jukes and Cantor 1969; Yang 1994a). Note that in (B), ML under the incorrect model (JC) is more efficient than ML under the correct model (JC+G).

The theory for ML in Appendix B applies generally to ML selection of nonnested models, whether one model (which may and may not be the true model) fits the data better than the others, judged by the K–L divergence to the true data-generating model. In particular, the theory applies to conventional phylogenetic reconstruction without the MSC model. For example, figure 5 applies the same prediction to simulation results on four-taxa trees from Yang (1997). Previously, Susko (2011) developed a large-sample approximation to the log-likelihood difference between two trees and to the probability that each tree will be the ML tree in the case of four-species without the molecular clock. It was assumed that the internal branch length in the tree is small and approaches 0 at the rate of $n^{-\frac{1}{2}}$ or faster when the number of sites *n* increases. In our analysis, we take the conventional approach of fixing the parameters when the data size increases.

We note that in problems of parameter estimation, the standard error for the parameter estimate or the width of the confidence interval typically decreases at the rate of $n^{-\frac{1}{2}}$, so that quadrupling the data size halves the interval. In contrast, the probability of recovering the best-fitting model approaches 1 much faster. As the probit transform of the error decreases linearly with $\sqrt{n}$, it will soon reach a point beyond which the precise error probability is of no practical significance: for example, $\Phi^{-1}(e)=-3$ means *e *=* *0.0013, while $\Phi^{-1}(e)=-5$ means $e=2.9\times 10^{-7}$. The different dynamics between model selection and parameter estimation when the data size grows is consistent with the fact that we tend to obtain extreme support for phylogenies inferred in large data sets (Yang and Zhu 2018).

### Implications of Our Study to Species Tree Methods

Although the species tree problem studied here is the simplest, it has the complexities of the general problem. Furthermore, we have represented all major species tree methods in our analysis. We expect ML to be asymptotically similar to Bayesian inference as both are full-data methods.

We have assumed the JC mutation model and the molecular clock. Our results are thus applicable to shallow species phylogenies and may not apply to distantly related species for which the JC model may be inadequate for multiple-hit correction and the molecular clock may be seriously violated. In the case of three species examined in this paper, concatenation and isml always produce the same species tree estimate. However, in more general settings with four or more species and when the clock is violated and unrooted trees are used, concatenation and isml are known to be different. In particular, concatenation (as well as 2-step) can be inconsistent (Roch and Steel 2015), while isml is a coalescent-aware method and is always consistent.

The isml method considered here is similar to SVDQuartets (Chifman and Kubatko 2014). Both are summary methods based on pooled site-pattern counts. SVDQuartets is sometimes described as a site pattern-based method (e.g., Kubatko 2019). This is not a helpful description. Site-pattern counts for different loci $\left(\{f_{ij}\}\right)$ are sufficient statistics under the model and carry the same amount of information as the sequence alignments at the same loci so that it makes no difference whether site patterns or sequences are used. Indeed virtually all methods involving likelihood calculation on sequences operate on site patterns instead of sites. Instead what matters is whether site patterns are pooled across loci. In the original data, the sites of the same locus share the same gene tree and the variation among loci provides information about parameters of the coalescent process such as the ancestral population sizes. Pooling sites across loci means that such information is lost (Shi and Yang 2018). As a result, the pooled site-pattern counts are unable to identify all parameters of the MSC model even if they can identify the species tree topology. Previously, Long and Kubatko (2018) found in simulations that SVDQuartets performed better in data sets of 600 coalescent-independent sites (m=600,n=1 in the notation of this paper) than in data of two genes each of 300 bp (m=2,n=300), and suggested that this is because “[t]he 600 sites observed from 600 distinct gene trees give independent genealogical information about the species tree, though indirectly, whereas the 300 sites for each of the two genes can give a reasonable indication of the individual gene trees, but still provide only two observed gene genealogies.” Our analysis suggests that this is not a correct interpretation. When the information in the data is used properly (as in the ML method), there is in fact more information in two genes each of 300 bp than in 600 independent sites (fig. 3*c*).

To understand the issue of parameter unidentifiability and the potential information loss for species tree estimation due to the pooling of sites across loci in SVDQuartets, consider the simple random-effects model:

(20) $$y_{ij}=\mu +\alpha_{i}+e_{ij},\;i=1,\ldots ,m;j=1,\ldots ,n,$$

where the treatment effect $\alpha_{i}∼N(0,\sigma_{a}^{2})$ and the error $e_{ij}∼N(0,\sigma_{e}^{2})$. Parameters in the model include the grand mean *μ* and the variance components $\sigma_{a}^{2}$ and $\sigma_{e}^{2}$. It is obvious that if there are no replications within treatment (*n *=* *1) or if the observations (*y_ij_*) are pooled across treatments, the between-treatment variation and within-treatment errors will be confounded so that $\sigma_{a}^{2}$ and $\sigma_{e}^{2}$ will not be identifiable even though *μ* still is. In species tree estimation, pooling site patterns across loci (as in isml and SVDQuartets) causes some parameters of the MSC model to become unidentifiable even though the species tree still is. This issue of information loss due to averaging over the whole genome may be even more serious for methods designed for data of single nucleotide polymorphisms (SNPs) (Leaché and Oaks 2017), such as snapp (Bryant et al. 2012), because the removal of constant sites in the SNP data causes further loss of information (even if the ascertainment bias is accounted for in the method).

An important difference between isml and SVDQuartets is that isml applies ML to the pooled site-pattern counts, whereas SVDQuartets uses a criterion based on linear invariants to avoid the ML optimization (Xu and Yang 2016). Use of a non-ML criterion is expected to lead to further reduction in efficiency, in addition to information loss due to the pooling of sites across loci (Chou et al. 2015; Xu and Yang 2016; Shi and Yang 2018).

The MSC model analyzed in this paper assumes free recombination among loci and no recombination between sites of the same locus. Data for such analysis are typically loosely linked short genomic segments that are far apart from each other so that recombination within a locus is rare, whereas different loci are nearly independent (e.g.,Takahata et al. 1995; Burgess and Yang 2008; Lohse et al. 2016). Both assumptions of free recombination among loci and no recombination within locus are expected to be violated in real data analysis, and the impact of within-locus recombination is of particular concern. The ML method considered in this paper assumes no recombination (with *r *=* *0), whereas isml (and SVDQuartets) assumes free recombination (r=∞). The relative performance of the methods will depend on the true recombination rate: ML may be expected to perform better than isml if *r* is close to 0, while isml may be superior if *r* is large. At very high recombination rates, it may even be possible for ML (assuming *r *=* *0) to be inconsistent since the method is similar to concatenation and merges sites of the same locus with different histories into one sequence. In contrast, isml is consistent for all values of *r*. Previously, Lanier and Knowles (2012) found in a computer simulation that species-tree estimation was robust to moderate levels of within-locus recombination (see also discussions in Edwards et al. [2016];Xu and Yang [2016]). It will be interesting to evaluate the relative performance of modern species-tree estimation methods (including isml and SVDQuartets) under realistic recombination rates.

## Materials and Methods

### Simulation

We use a challenging species tree with parameters $\tau_{0}=0.02,\;\tau_{1}=0.019$, $\theta_{0}=0.01$, and $\theta_{1}=0.05$ (fig. 1*a*). A C program is written to simulate gene trees and sequence alignments for the case of three species/sequences, under the JC model (Jukes and Cantor 1969) with the clock. To simulate the gene tree and the sequence alignment for each locus, we generate an exponential coalescent waiting time (*s*_1_) with mean $\theta_{1}/2$. If $s_{1}<\tau_{1}$, the gene tree is $G_{1a}$, and another exponential waiting time *s*_0_ is generated with mean $\theta_{0}/2$ to get $t_{0}=\tau_{0}+s_{0}$ and *t*_1_ = *s*_1_. If $s_{1}>\tau_{1}$, the gene tree is one of $G_{1b},G_{2},G_{3}$, chosen at random, and two coalescent waiting times (*s*_1_ and *s*_0_) are generated with means $\theta_{0}/6$ and $\theta_{0}/2$, respectively, so that $t_{1}=\tau_{0}+s_{1}$ and $t_{0}=t_{1}+s_{0}$ (fig. 1*b*). The gene tree and node ages $\left(t_{0},t_{1}\right)$ are then used to calculate the site-pattern probabilities for the locus (eq. 3), and the site-pattern counts are generated from multinomial sampling (eq. 4). Each data set consists of *m* loci with the sequence length of *n* sites. We use a large number of replicates (typically $R=10^{6}$ or 10^8^) so that sampling errors due to a limited number of replicates is not a concern. Species tree estimation by concatenation (=isml) and 2-step is done by counting site patterns.

For the ML method (eq. 5), we used the simulation program MCcoal, which is part of the bpp program (Yang 2015), to simulate the gene trees and sequence alignments. The data are then analyzed using the ML program 3s (Yang 2002; Dalquen et al. 2017). The JC model is used to simulate and analyze data.

## Supplementary Material

Supplementary data are available at *Molecular Biology and Evolution* online.

## Supplementary Material

msab009_Supplementary_Data

## Figures and Tables

**Fig. 6.**
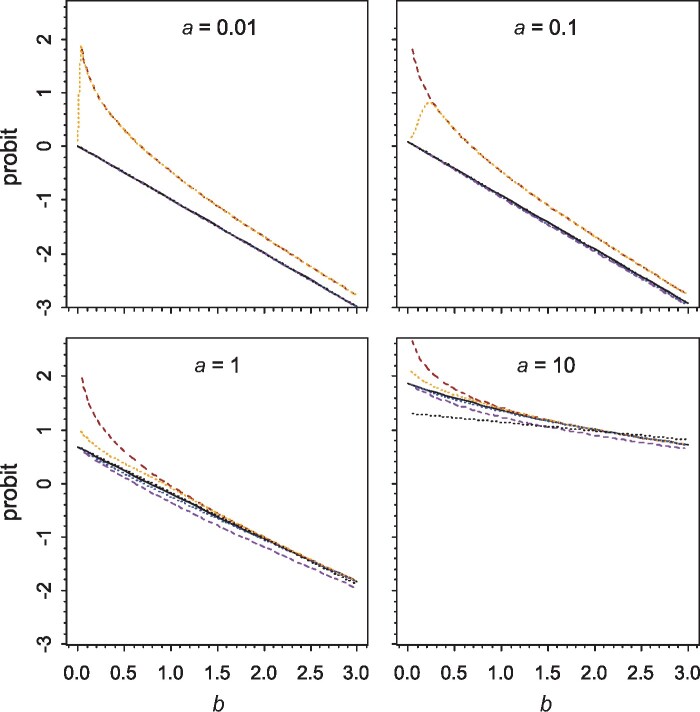
Probit of error, $\Phi^{-1}(\zeta )$, plotted against *b* for different values of *a*. Six methods for calculating *ζ* are shown. The first five are, from top to bottom, $\zeta_{U1}$ (brown dashed line), $\zeta_{U2}$ (orange dotted, with *k* = 2 in eq. A27), Exact (black solid line), $\zeta_{L2}$ (blue dotted), and $\zeta_{L1}$ (purple dashed). Equation (6) (black dotted) is included as well.

**Fig. 7.**
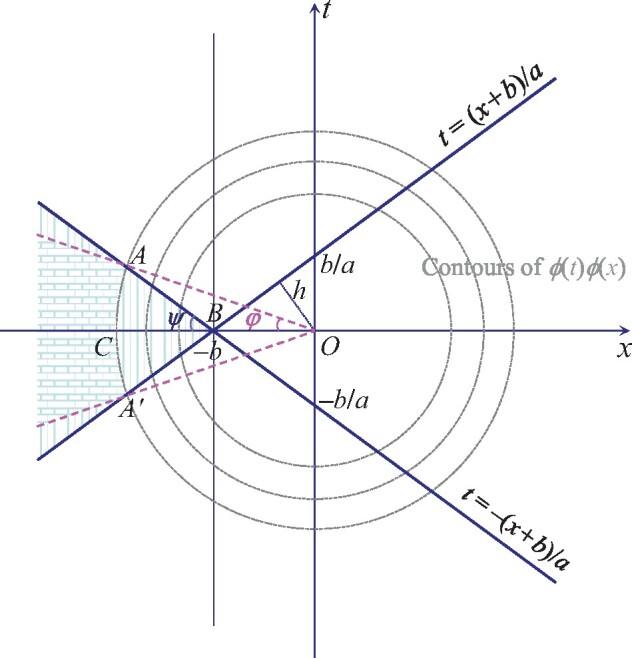
The areas of integration for integrals in Equations (A11) and (A12). The two angles are $\psi ={\;\text{tan}\;}^{-1}\frac{1}{a}$ and $\varphi ={\;\text{tan}\;}^{-1}\frac{1}{ka}$, *k *>* *1, with $\varphi <\psi <\frac{\pi }{2}$. The integral over the half-plane x<−b is Φ(−b), whereas the integral over the half-plane t>(x+b)/a is $S=\Phi (-h)=P\{{\bar{z}}_{1}<{\bar{z}}_{2}\}$. The integral over the sector ABA′ (the shaded area) is $2A=P\{{\bar{z}}_{1}<{\bar{z}}_{2},{\bar{z}}_{1}<{\bar{z}}_{3}\}$. This is smaller than $\Phi (-b)\cdot \psi /\frac{\pi }{2}$ and greater than the integral over the area shaded with the brick pattern: these give the bounds ($\zeta_{L2},\zeta_{U2}$) in Appendix A. The purple dashed lines are t=x/(ka) and t=−x/(ka). They cross the blue lines at *A* and A′, with the length of the line segment *OA* to be $r=\frac{b\sqrt{a^{2}k^{2}+1}}{a(k-1)}$. Note that the integral over the circle $x^{2}+t^{2}<r^{2}$ is $1-e^{-\frac{1}{2}r^{2}}$.

## Appendix A. Proof of Theorem 1

(a) Define the random variable:

(A1) $$\begin{array}{c} y={\bar{z}}_{1}-\max({\bar{z}}_{2},{\bar{z}}_{3})={\bar{z}}_{1}-\frac{1}{2}({\bar{z}}_{2}+{\bar{z}}_{3})-\frac{1}{2}|{\bar{z}}_{2}-{\bar{z}}_{3}|\\ =y_{1}-|y_{2}|, \end{array}$$

where $y_{1}={\bar{z}}_{1}-\frac{1}{2}({\bar{z}}_{2}+{\bar{z}}_{3})∼N(\Delta \mu ,s_{1}^{2}/m)$ and $y_{2}=\frac{1}{2}({\bar{z}}_{2}-{\bar{z}}_{3})∼N(0,s_{2}^{2}/m)$, with

(A2) $$\begin{array}{cc} \frac{1}{m}s_{1}^{2} & =V({\bar{z}}_{1})+\frac{1}{4}V({\bar{z}}_{2}+{\bar{z}}_{3})-2\text{Cov}({\bar{z}}_{1},{\bar{z}}_{2})\\ & =\frac{1}{m}[\sigma_{1}^{2}+\frac{1}{2}(\sigma_{2}^{2}+\sigma_{23})-2\sigma_{12}],\\ \frac{1}{m}s_{2}^{2} & =\frac{1}{4}V{\left({\bar{z}}_{2}-{\bar{z}}_{3}\right)}^{2}=\frac{1}{2m}(\sigma_{2}^{2}-\sigma_{23}). \end{array}$$

Here, we treat ${\bar{z}}_{1},{\bar{z}}_{2}$ and ${\bar{z}}_{3}$ as normal variables, according to the central limit theorem as m→∞. As $\text{Cov}(y_{1},y_{2})=0$ and both *y*_1_ are *y*_2_ are normal variables, they are independent. Then,

(A3) $$\begin{array}{cc} \zeta & =P\{y_{1}<|y_{2}|\}\\ & =P\{y_{2}<0,y_{1}<-y_{2}\}+P\{y_{2}>0,y_{1}<y_{2}\}\\ & =2P\{y_{2}>0,y_{1}<y_{2}\}\\ & =2\int_{0}^{\infty }\phi (y_{2};0,\frac{1}{m}s_{2}^{2})\Phi (\frac{y_{2}-\Delta \mu }{s_{1}/\sqrt{m}})dy_{2}\\ & =2\int_{0}^{\infty }\phi (t)\Phi (at-b)dt, \end{array}$$

where $a=s_{2}/s_{1},\;b=\Delta \mu \sqrt{m}/s_{1}$, and $\phi (x;\mu ,\sigma^{2})$ is the probability density function (PDF) for $N(\mu ,\sigma^{2})$, whereas ϕ(x) is the PDF for N(0,1). The last integral has been studied by Yang and Rodríguez (2013, SI) in a different context and can be written as:

(A4) $$\zeta =\frac{1}{\pi }\int_{-\frac{\pi }{2}}^{{\;\text{tan}\;}^{-1}a}\;\text{exp}\;\left\{-\frac{b^{2}}{2{\left(\text{sin}\;\theta -a\;\text{cos}\;\theta \right)}^{2}}\right\}d\theta ,$$

or, by letting t=a−tan θ, with dθ = −1/[(*t*−*a*)^2+1^] d*t*, as:

(A5) $$\zeta =\frac{1}{\pi }\int_{0}^{\infty }\frac{1}{{\left(t-a\right)}^{2}+1}e^{-\frac{b^{2}[{\left(t-a\right)}^{2}+1]}{2t^{2}}}dt.$$

Equations (A4) and (A5) can be calculated using Gaussian quadrature and match direct calculations using the CDF for the bivariate normal distribution for $\left({\bar{z}}_{1}-{\bar{z}}_{2},{\bar{z}}_{1}-{\bar{z}}_{3}\right)$. When Δμ=0, we have *b *=* *0 and:

(A6) $$\zeta =2\int_{0}^{\infty }\phi (t)\Phi (at)dt=\frac{1}{2}+\frac{1}{\pi }{\;\text{tan}\;}^{-1}a.$$

In the symmetrical case of $\Delta \mu =0,\;\sigma_{1}^{2}=\sigma_{2}^{2}$, and $\sigma_{12}=\sigma_{23}$ (with $a=\frac{1}{\sqrt{3}}$, *b *=* *0), this gives $\frac{1}{2}+\frac{1}{\pi }{\;\text{tan}\;}^{-1}(\frac{1}{\sqrt{3}})=\frac{2}{3}$, as expected. In this case the three variables ${\bar{z}}_{1},{\bar{z}}_{2}$ and ${\bar{z}}_{3}$ have the same probability of being the greatest so that the error is $\frac{2}{3}$.

To avoid numerical integration, we note that $y_{2}∼N(0,\frac{1}{2m}(\sigma_{2}^{2}-\sigma_{23}))$, and $\left|y_{2}\right|$ is a folded normal variable with mean and variance:

(A7) $$\begin{array}{cc} E(|y_{2}|) & =\sqrt{\frac{1}{m\pi }(\sigma_{2}^{2}-\sigma_{23})},\\ V(|y_{2}|) & =(\frac{1}{2m}-\frac{1}{m\pi })(\sigma_{2}^{2}-\sigma_{23}). \end{array}$$

Thus,

(A8) $$\begin{array}{cc} E(y) & =\Delta \mu -\sqrt{\frac{1}{m\pi }(\sigma_{2}^{2}-\sigma_{23})}.\\ V(y) & =V({\bar{z}}_{1})+\frac{1}{4}V({\bar{z}}_{2}+{\bar{z}}_{3})+\frac{1}{4}V(|{\bar{z}}_{2}-{\bar{z}}_{3}|)\\ & -\;\text{Cov}({\bar{z}}_{1},{\bar{z}}_{2}+{\bar{z}}_{3})-\text{Cov}({\bar{z}}_{1},|{\bar{z}}_{2}-{\bar{z}}_{3}|)\\ & +\frac{1}{2}\text{Cov}({\bar{z}}_{2}+{\bar{z}}_{3},|{\bar{z}}_{2}-{\bar{z}}_{3}|)\\ & =V({\bar{z}}_{1})+\frac{1}{4}V({\bar{z}}_{2}+{\bar{z}}_{3})+\frac{1}{4}V(|{\bar{z}}_{2}-{\bar{z}}_{3}|)\\ & -\;2\text{Cov}({\bar{z}}_{1},{\bar{z}}_{2})-\text{Cov}({\bar{z}}_{1},|{\bar{z}}_{2}-{\bar{z}}_{3}|)\\ & +\;\text{Cov}({\bar{z}}_{2},|{\bar{z}}_{2}-{\bar{z}}_{3}|). \end{array}$$

We have,

(A9) $$\begin{array}{c} V({\bar{z}}_{2}+{\bar{z}}_{3})+V(|{\bar{z}}_{2}-{\bar{z}}_{3}|)=E{\left({\bar{z}}_{2}+{\bar{z}}_{3}\right)}^{2}+E{\left({\bar{z}}_{2}-{\bar{z}}_{3}\right)}^{2}\\ -E^{2}({\bar{z}}_{2}+{\bar{z}}_{3})-E^{2}(|{\bar{z}}_{2}-{\bar{z}}_{3}|)\\ =4E({\bar{z}}_{2}^{2})-4\mu_{2}^{2}-\frac{4}{m\pi }(\sigma_{2}^{2}-\sigma_{23})\\ =\frac{4}{m}\sigma_{2}^{2}-\frac{4}{m\pi }(\sigma_{2}^{2}-\sigma_{23}),\\ \text{Cov}({\bar{z}}_{1},|{\bar{z}}_{2}-{\bar{z}}_{3}|)=0,\\ \text{Cov}({\bar{z}}_{2},|{\bar{z}}_{2}-{\bar{z}}_{3}|)=0. \end{array}$$

Collecting all terms in equation (A8), we get

(A10) $$V(y)=\frac{1}{m}\left[\sigma_{1}^{2}-2\sigma_{12}+\sigma_{2}^{2}-\frac{1}{\pi }(\sigma_{2}^{2}-\sigma_{23})\right]$$

If we assume that *y* is approximately normally distributed, as in Zharkikh and Li (1992) and Yang (1996), then equation (6) follows. Note that equation (6) can also be written as $\zeta_{N}=\Phi \left(\frac{-b+a\sqrt{2/\pi }}{\sqrt{1+a^{2}(1-\frac{2}{\pi })}}\right)$. Because $\left|y_{2}\right|$ has a folded normal distribution and is not a normal variable, the error of approximation of equation (6) does not approach zero when m→∞. For instance, in the symmetrical case ($\Delta \mu =0,\;\sigma_{1}^{2}=\sigma_{2}^{2}$, and $\sigma_{12}=\sigma_{23}$), equation (6) gives $\Phi (\frac{1}{\sqrt{2\pi -1}})=0.66824$, not $\frac{2}{3}$. This level of accuracy is acceptable for our calculations for finite *m* in this paper, as the precise value of the error is unimportant if the error is nearly zero, but equation (6) may not give the correct asymptotic error rate when m→∞ (fig. 6, *a *=* *10).

(b) To study the asymptotic behavior of the error probability *ζ* when m→∞, we derive bounds on *ζ*. From equation (A3),

(A11) $$\begin{array}{cc} \zeta & =2\int_{0}^{\infty }\phi (t)\int_{-\infty }^{at-b}\phi (x)dxdt\\ & =2\int_{-\infty }^{\infty }\int_{-\infty }^{at-b}\phi (t)\phi (x)dxdt-2\int_{-\infty }^{0}\int_{-\infty }^{at-b}\phi (t)\phi (x)dxdt\\ & =2S-2A, \end{array}$$

where the first integral is S=Φ(−h), with $h=\frac{b}{\sqrt{1+a^{2}}}=\frac{\Delta \mu \sqrt{m}}{\sqrt{\sigma_{1}^{2}-2\sigma_{12}+\sigma_{2}^{2}}}$ to be the distance from the origin (0, 0) to the line x=at−b (fig. 7), and the second integral is:

(A12) $$\begin{array}{cc} 2A & =2\int_{-\infty }^{0}\int_{-\infty }^{at-b}\phi (t)\phi (x)dxdt\\ & =\int_{-\infty }^{-b}\phi (x)\int_{(x+b)/a}^{-(x+b)/a}\phi (t)dtdx. \end{array}$$

By considering the area of integration (fig. 7), it is obvious that:

(A13) $$0<2A\leq \Phi (-b)[1-\frac{2}{\pi }{\;\text{tan}\;}^{-1}a],$$

where the equality holds when *b *=* *0. Let,

$$\zeta_{L2}=2\Phi (-h)-\Phi (-b)[1-\frac{2}{\pi }{\;\text{tan}\;}^{-1}a].$$

As Φ(−b)<Φ(−h), we have:

(A14) $$\zeta_{L2}>\Phi (-h)[1+\frac{2}{\pi }{\;\text{tan}\;}^{-1}a]\equiv \zeta_{L1},$$

or

(A15) $$\Phi (-h)\leq \zeta_{L1}\leq \zeta_{L2}\leq \zeta <2\Phi (-h)\equiv \zeta_{U1},$$

as in equation (7). The equality in the lower bounds is achieved at b=0. Note that the bounds apply to all *a *>* *0 and *b *>* *0. We use the bounds ($\zeta_{L1},\zeta_{U1}$) in Theorem 1 and in the calculation of table 1. The width of the interval is $\Phi (-h)[1-\frac{2}{\pi }{\;\text{tan}\;}^{-1}a]\leq \Phi (-h)\leq \zeta$, so that using any value inside the interval as the estimate will give an error of approximation that is smaller than the error probability *ζ*.

Note that the bounds Φ(−h)<ζ<2Φ(−h) are also given by the definition $\zeta =P\{{\bar{z}}_{1}<{\bar{z}}_{2}\cup {\bar{z}}_{1}<{\bar{z}}_{3}\}$, since

(A16) $$P({\bar{z}}_{1}<{\bar{z}}_{2})<\zeta <P({\bar{z}}_{1}<{\bar{z}}_{2})+P({\bar{z}}_{1}<{\bar{z}}_{3})=2P({\bar{z}}_{1}<{\bar{z}}_{2}),$$

with $P({\bar{z}}_{1}<{\bar{z}}_{2})=\Phi (-h)$.

Next we consider the upper bound in equation (A15) when *h* or *b* is large. Note that:

(A17) $$\begin{array}{cc} \Phi (-h) & =\int_{h}^{\infty }\frac{1}{\sqrt{2\pi }}e^{-y^{2}/2}dy\\ & =\int_{0}^{\infty }\frac{1}{\sqrt{2\pi }}e^{-\frac{1}{2}{\left(x+h\right)}^{2}}dx\\ & =\frac{1}{\sqrt{2\pi }}e^{-h^{2}/2}\int_{0}^{\infty }e^{-(hx+\frac{1}{2}x^{2})}dx\\ & =\frac{1}{h\sqrt{2\pi }}e^{-h^{2}/2}\int_{0}^{\infty }e^{-t}e^{-\frac{1}{2h^{2}}t^{2}}dt\\ & =\frac{1}{h\sqrt{2\pi }}e^{-h^{2}/2}B, \end{array}$$

where $B=\int_{0}^{\infty }e^{-t}e^{-\frac{1}{2h^{2}}t^{2}}dt<\int_{0}^{\infty }e^{-t}dt=$ 1. For large *h*,

(A18) $$\begin{array}{cc} B & >\int_{0}^{\sqrt{h}}e^{-t}e^{-\frac{1}{2h^{2}}t^{2}}dt>e^{-\frac{1}{2h}}\int_{0}^{\sqrt{h}}e^{-t}dt\\ & =e^{-\frac{1}{2h}}(1-e^{-\sqrt{h}})=1-\frac{1}{2h}+o\left(\frac{1}{h}\right). \end{array}$$

Thus, for large *h*, Φ(−h) is bounded by:

(A19) $$\left(1-\frac{1}{2h}+o(\frac{1}{h})\right)\frac{1}{h\sqrt{2\pi }}e^{-h^{2}/2}<\Phi (-h)<\frac{1}{h\sqrt{2\pi }}e^{-h^{2}/2},$$

or

(A20) $$\Phi (-h)=\frac{1}{h\sqrt{2\pi }}e^{-h^{2}/2}+O\left(\frac{1}{h^{2}}e^{-h^{2}/2}\right).$$

Let ɛ>0 such that Φ(−(h+ɛ))=αΦ(−h) for 0<α<1; in other words, *ɛ* is the offset at the probit level to reduce the probability by a fraction. From equation (A20),

(A21) $$\begin{array}{c} \frac{1}{(h+ɛ)\sqrt{2\pi }}e^{-\frac{1}{2}(h^{2}+2ɛh+{ɛ}^{2})}+O(\frac{1}{{\left(h+ɛ\right)}^{2}}e^{-\frac{1}{2}{\left(h+ɛ\right)}^{2}})\\ =\frac{\alpha }{h\sqrt{2\pi }}e^{-\frac{1}{2}h^{2}}+O(\frac{1}{h^{2}}e^{-\frac{1}{2}h^{2}}). \end{array}$$

Thus,

(A22) $$\frac{1}{h+ɛ}e^{-\frac{1}{2}(h^{2}+2ɛh+{ɛ}^{2})}=\frac{\alpha }{h}e^{-\frac{1}{2}h^{2}}+O(\frac{1}{h^{2}}e^{-\frac{1}{2}h^{2}}),$$

which gives $ɛ=-\frac{1}{h}\text{log}\;\alpha +o(\frac{1}{h})$ or

(A23) $$\Phi \left(-h+\frac{1}{h}\text{log}\;\alpha +o(\frac{1}{h})\right)=\alpha \Phi (-h).$$

In particular, for $\alpha =\frac{1}{2}$, we have:

(A24) $$\Phi \left(-\left(h+\frac{1}{h}\text{log}\;2+o(\frac{1}{h})\right)\right)=\frac{1}{2}\Phi (-h).$$

Thus, for large *h*, we have:

(A25) $$2\Phi (-h)=\Phi (-h+\frac{1}{h}\text{log}\;2+o(\frac{1}{h})),$$

as in equation (7). It may be noteworthy that for large *h*, a very small change at the probit level, of about $\frac{1}{h}\text{log}\;2$, changes the probability by a factor of 2.

A tighter lower bound for 2 *A* than zero of equation (A13) is:

(A26) $$2A>\frac{\varphi }{\pi }\text{exp}\;\left\{-\frac{b^{2}(a^{2}k^{2}+1)}{2a^{2}{\left(k-1\right)}^{2}}\right\},$$

where $\varphi ={\;\text{tan}\;}^{-1}\frac{1}{ka}$ with *k *>* *1 (fig. 7). Thus, we have a tighter pair of bounds on *ζ*,

(A27) $$\begin{array}{c} 2\Phi (-h)-\Phi (-b)[1-\frac{2}{\pi }{\;\text{tan}\;}^{-1}a]\leq \zeta <\\ 2\Phi (-h)-\frac{1}{\pi }{\;\text{tan}\;}^{-1}\frac{1}{ka}\text{exp}\{-\frac{b^{2}(a^{2}k^{2}+1)}{2a^{2}{\left(k-1\right)}^{2}}\}, \end{array}$$

where *k *>* *1. We write this pair of bounds as $\zeta_{L2}<\zeta <\zeta_{U2}$. We have $\Phi (-b)\leq \Phi (-h)\leq \zeta_{L1}\leq \zeta_{L2}\leq \zeta <\zeta_{U2}<\zeta_{U1}=2\Phi (-h)$. These bounds, as well as the exact value and equation (6), are plotted against *b* in figure 6 for a=0.01,0.1,1 and 10.

## Appendix B. The asymptotics of ML species tree estimation

The proof below borrows heavily from White (1982), Dawid (2011), and Yang and Zhu (2018). Let $S_{j},j=1,2,3$ be the three species trees with parameters $\theta_{j}$. Note that *S*_1_ is the true model, while *S*_2_ and *S*_3_ are mis-specified models. Let the data at *m* loci be $x=\{x_{i}\},i=1,\ldots ,m$. The log-likelihood function is $\ell_{j}(\theta_{j})=\text{log}\;f(x|S_{j},\theta_{j})$. We also define $l_{j}(\theta_{j})=\text{log}\;f(x|S_{j},\theta_{j})$ for one data point (that is, site-pattern counts at any single locus), $x\equiv (x_{i0},x_{i1},x_{i2},x_{i3},x_{i4})$. When the number of loci m→∞, the MLE ${\hat{\theta }}_{j}\to \theta_{j}^{*}$. We assume that both ${\hat{\theta }}_{j}$ and $\theta_{j}^{*}$ are inner points in the parameter space. Whether ${\hat{\theta }}_{j}$ is inside the parameter space or at its boundary should not affect the asymptotic rate of convergence. Here, $\theta_{1}^{*}$ for the true species tree *S*_1_ is the true parameter value, whereas $\theta_{2}^{*}$ for *S*_2_ (as well as $\theta_{3}^{*}$ for *S*_3_) is the *pseudotrue parameter value*, which minimizes the Kullback–Leibler distance from the misspecified model *S*_2_ to the true model *S*_1_.

(A28) $$\begin{array}{cc} D_{12}=\int f(x|S_{1},\theta_{1}^{*})\;\text{log}\;\frac{f(x|S_{1},\theta_{1}^{*})}{f(x|S_{2},\theta_{2}^{*})}\mathrm{dx}\\ & \;=E\{l_{1}(\theta_{1}^{*})-(l_{2}(\theta_{2}^{*})\}, \end{array}$$

where the expectation is over the true distribution $f(x|S_{1},\theta_{1}^{*})$. *D*_13_ is defined similarly, with $D_{13}=D_{12}$.

We consider the log-likelihood ratio, $\ell_{j}(\hat{\theta })-\ell_{j}(\theta^{*})$, given the data (*x*) for any of the species tree *j*. We drop the subscript *j* for clarity. As in White (1982) and Dawid (2011), we define two matrices:

(A29) $$\begin{array}{cc} I(\theta ) & =E\{\nabla \;\text{log}\;f(x|\theta )\cdot \nabla \;\text{log}\;f{\left(x|\theta \right)}^{T}\}\\ & =E\{l\prime (\theta ){\left(l^{\prime }(\theta )\right)}^{T}\},\\ J(\theta ) & =E\{-\nabla^{2}\;\text{log}\;f(x|\theta )\}=E\{-l\prime \prime (\theta )\}, \end{array}$$

(A29)

where the superscript *T* stands for transpose and where the expectation is over the true distribution, and ∇ and $\nabla^{2}$ are the first and second derivatives with respect to θ.

Apply Taylor expansion to the log likelihood around the MLE $\hat{\theta }$:

(A30) $$\ell (\theta )\approx \ell (\hat{\theta })+\ell \prime (\hat{\theta })(\theta -\hat{\theta })+\frac{1}{2}{\left(\theta -\hat{\theta }\right)}^{T}\ell \prime \prime (\hat{\theta })(\theta -\hat{\theta }),$$

where both the gradient and the Hessian are evaluated at the MLE ( $\hat{\theta }$), with $\ell \prime (\hat{\theta })=0$. Setting $\theta =\theta^{*}$, we have:

(A31) $$\ell (\hat{\theta })\approx \ell (\theta^{*})+\frac{1}{2}{\left(\hat{\theta }-\theta^{*}\right)}^{T}(-\ell \prime \prime (\hat{\theta }))(\hat{\theta }-\theta^{*}).$$

Apply Taylor expansion to the derivative ℓ′(θ) around the MLE $\hat{\theta }$ and let $\theta =\theta^{*}$, and we have:

(A32) $$\ell \prime (\theta )\approx \ell \prime \prime (\hat{\theta })(\theta -\hat{\theta }),$$

and

(A33) $$\hat{\theta }-\theta^{*}\approx -\ell \prime \prime {\left(\hat{\theta }\right)}^{-1}\ell \prime (\theta^{*}).$$

Each of $\ell \prime (\hat{\theta })$ and $\ell \prime \prime (\hat{\theta })$ is a sum of *m* i.i.d. elements. When $m\to \infty ,-\ell \prime \prime (\hat{\theta })\approx mE\{-l\prime \prime (\theta^{*})\}=mJ^{*}$, with $J^{*}=J(\theta^{*})$ (eq. A29). Furthermore,

(A34) $$\begin{array}{cc} E\{\ell \prime (\theta^{*})\} & =0,\\ V\{\ell \prime (\theta^{*})\} & =mV\{l\prime (\theta^{*})\}=mI^{*}, \end{array}$$

where $I^{*}=I(\theta^{*})$ (eq. A29). Thus,

(A35) $$\sqrt{m}(\hat{\theta }-\theta^{*})\overset{P}{\underset{\;}{\to }}N\left(0,{\left(J^{*-1}\right)}^{T}I^{*}(J^{*-1})\right).$$

Thus, $\hat{\theta }=\theta^{*}+O_{p}(m^{-1/2})$. Equation (A31) becomes:

(A36) $$\begin{array}{c} \ell (\hat{\theta })\approx \ell (\theta^{*})+\frac{1}{2}{\left\{\sqrt{m}(\hat{\theta }-\theta^{*})\right\}}^{T}J^{*}\{\sqrt{m}(\hat{\theta }-\theta^{*})\} \end{array}$$

$$=\ell (\theta^{*})+O_{p}(1).$$

Equations (A29–A36) apply to all three species trees. In the case of *S*_1_ (the true model), $J^{*}=I^{*}$, the Fisher information matrix, and $\ell (\hat{\theta })-\ell (\theta^{*})∼\frac{1}{2}\chi_{d}^{2}$. For *S*_2_ or *S*_3_, $\ell (\hat{\theta })-\ell (\theta^{*})$ is a quadratic form of normal variates and is a mixture of noncentral $\chi^{2}$ variables with mean $\frac{1}{2}\text{tr}(I^{*}J^{*-1})$ and variance $\frac{1}{2}\text{tr}({\left(I^{*}J^{*-1}\right)}^{2})$, both of *O*(1).

Now consider using ${\bar{z}}_{j}\equiv \frac{1}{m}\ell_{j}({\hat{\theta }}_{j})$, *j *=* *1, 2, 3, to compare species trees *S*_1_, *S*_2_, and *S*_3_. We have:

(A37) $$\begin{array}{c} E({\bar{z}}_{j})\approx E(l_{j}(\theta_{j}^{*}))\equiv \mu_{j},\\ V({\bar{z}}_{j})\approx \frac{1}{m}V(l_{j}(\theta_{j}^{*}))\equiv \frac{1}{m}\sigma_{jj},\\ \text{Cov}({\bar{z}}_{j},{\bar{z}}_{k})\approx \frac{1}{m}\text{Cov}(l_{j}(\theta_{j}^{*}),l_{k}(\theta_{k}^{*}))\equiv \frac{1}{m}\sigma_{jk}. \end{array}$$

Thus, when the number of loci $m\to \infty ,\;\{{\bar{z}}_{j}\}=\{\frac{1}{m}\ell_{j}({\hat{\theta }}_{j})\}$ have means $\left(\mu_{1},\mu_{2},\mu_{2}\right)$ and variance/covariance matrix $\frac{1}{m}\Sigma$, where $\Sigma =\{\sigma_{jk}\}$ is *O*(1) and independent of *m*. The error of the ML method, $P\{\ell_{1}({\hat{\theta }}_{1})>\max(\ell_{2}({\hat{\theta }}_{2}),\ell_{3}({\hat{\theta }}_{3}))\}=P\{{\bar{z}}_{1}>\max({\bar{z}}_{2},{\bar{z}}_{3})\}$, is then given by Theorem 1 as equation (11).
