# Supplementary material for: Complexity of the simplest species tree problem
Source: Mol Biol Evol. 2021 Jan 25;38(9):3993–4009. doi: 10.1093/molbev/msab009 (PMC8382899; doi:10.1093/molbev/msab009)
Supplement: msab009_Supplementary_Data [file msab009_supplementary_data.pdf]

Table S1. Variances-covariance matrix of site-pattern frequencies (f_{jk}) among loci (with n sites per locus)

pattern	0 : *xxx*	1 : *xxy*	2 : *yxx*	3 : *xyx*	4 : *xyz*
mean ($\bar{p}_j$)	0.92831926	0.023777106	0.023372801	0.023372801	0.001158033
$n = 1$					
0: xxx	0.0665371525	−0.0220737114	−0.0216961587	−0.0216906657	−0.00107661674
1: xxy	−0.0220737114	0.0232125909	−0.000555721922	−0.000555581223	$-2.7576288 \times 10^{-5}$
2: yxx	−0.0216961587	−0.000555721922	0.022825064	−0.000546078464	$-2.71046183 \times 10^{-5}$
3: xyx	−0.0216906657	−0.000555581223	−0.000546078464	0.0228194232	$-2.70977559 \times 10^{-5}$
4: xyz	−0.00107661674	$-2.7576288 \times 10^{-5}$	$-2.71046183 \times 10^{-5}$	$-2.70977559 \times 10^{-5}$	0.00115839534
$n = 2$					
0: xxx	0.0333193524	−0.0110518301	−0.010863736	−0.0108646269	−0.000539159418
1: xxy	−0.0110518301	0.0116302167	−0.000282670114	−0.000282578449	$-1.31380675 \times 10^{-5}$
2: yxx	−0.010863736	−0.000282670114	0.0114355946	−0.000276246353	$-1.2942121 \times 10^{-5}$
3: xyx	−0.0108646269	−0.000282578449	−0.000276246353	0.0114364545	$-1.30027622 \times 10^{-5}$
4: xyz	−0.000539159418	$-1.31380675 \times 10^{-5}$	$-1.2942121 \times 10^{-5}$	$-1.30027622 \times 10^{-5}$	0.000578242368
$n = 10$					
0: xxx	0.00674696401	−0.00223806143	−0.00219929448	−0.00219926798	−0.000110340106
1: xxy	−0.00223806143	0.00236735227	$-6.38114276 \times 10^{-5}$	$-6.36625594 \times 10^{-5}$	$-1.81685014 \times 10^{-6}$
2: yxx	−0.00219929448	$-6.38114276 \times 10^{-5}$	0.00232577673	$-6.08615665 \times 10^{-5}$	$-1.80925447 \times 10^{-6}$
3: xyx	−0.00219926798	$-6.36625594 \times 10^{-5}$	$-6.08615665 \times 10^{-5}$	0.00232560997	$-1.81786191 \times 10^{-6}$
4: xyz	−0.000110340106	$-1.81685014 \times 10^{-6}$	$-1.80925447 \times 10^{-6}$	$-1.81786191 \times 10^{-6}$	0.000115784072
$n = 100$					
0: xxx	0.000767781846	−0.000254691861	−0.000249506713	−0.000249726749	$-1.38565232 \times 10^{-5}$
1: xxy	−0.000254691861	0.000282913562	$-1.45054073 \times 10^{-5}$	$-1.44492196 \times 10^{-5}$	$7.32926136 \times 10^{-7}$
2: yxx	−0.000249506713	$-1.45054073 \times 10^{-5}$	0.000275629759	$-1.23445851 \times 10^{-5}$	7.2694645×10^{-7}
3: xyx	−0.000249726749	$-1.44492196 \times 10^{-5}$	$-1.23445851 \times 10^{-5}$	0.000275789815	$7.30738276 \times 10^{-7}$
4: xyz	$-1.38565232 \times 10^{-5}$	$7.32926136 \times 10^{-7}$	7.2694645×10^{-7}	$7.30738276 \times 10^{-7}$	$1.16659123 \times 10^{-5}$
$n = 1000$					
0: xxx	0.000169701052	$-5.65782078 \times 10^{-5}$	$-5.44757242 \times 10^{-5}$	$-5.44615584 \times 10^{-5}$	$-4.18556146 \times 10^{-6}$
1: xxy	$-5.65782078 \times 10^{-5}$	$7.46936699 \times 10^{-5}$	$-9.51058048 \times 10^{-6}$	$-9.60077773 \times 10^{-6}$	$9.95896135 \times 10^{-7}$
2: yxx	$-5.44757242 \times 10^{-5}$	$-9.51058048 \times 10^{-6}$	$7.05311669 \times 10^{-5}$	$-7.5138745 \times 10^{-6}$	$9.69012304 \times 10^{-7}$
3: xyx	$-5.44615584 \times 10^{-5}$	$-9.60077773 \times 10^{-6}$	$-7.5138745 \times 10^{-6}$	$7.06080886 \times 10^{-5}$	$9.68122061 \times 10^{-7}$
4: xyz	$-4.18556146 \times 10^{-6}$	$9.95896135 \times 10^{-7}$	$9.69012304 \times 10^{-7}$	$9.68122061 \times 10^{-7}$	$1.25253095 \times 10^{-6}$
$n = \infty$					
0: xxx	0.000103268649	$-3.42669472 \times 10^{-5}$	$-3.29431183 \times 10^{-5}$	$-3.29485916 \times 10^{-5}$	$-3.10999147 \times 10^{-6}$
1: xxy	$-3.42669472 \times 10^{-5}$	5.1215364×10^{-5}	$-8.98272353 \times 10^{-6}$	$-8.98309369 \times 10^{-6}$	$1.01740041 \times 10^{-6}$
2: yxx	$-3.29431183 \times 10^{-5}$	$-8.98272353 \times 10^{-6}$	$4.78926166 \times 10^{-5}$	$-6.96552523 \times 10^{-6}$	$9.98750549 \times 10^{-7}$
3: xyx	$-3.29485916 \times 10^{-5}$	$-8.98309369 \times 10^{-6}$	$-6.96552523 \times 10^{-6}$	4.7898289×10^{-5}	$9.98921539 \times 10^{-7}$
4: xyz	$-3.10999147 \times 10^{-6}$	$1.01740041 \times 10^{-6}$	$9.98750549 \times 10^{-7}$	$9.98921539 \times 10^{-7}$	$9.49189765 \times 10^{-8}$

Note.— The parameter values used are $(\tau_0, \tau_1, \theta_0, \theta_1)$ = (0.02, 0.019, 0.01, 0.05). The means are calculated using eq. 13, confirmed by simulation. The variances at $n = \infty$ are estimated by simulating gene trees with coalescent times and calculating p_j (eq. 3). Those for other n are estimated by simulating gene trees, calculating site-pattern probabilities p_j (eq. 3), and then using them to sample the site-pattern counts from the multinomial distribution (eq. 4). The number of replicates ranges from $R = 10^6$ to 5×10^9.
